# Complex genetic encoding of the hepatitis B virus on-drug persistence

**DOI:** 10.1038/s41598-020-72467-9

**Published:** 2020-09-23

**Authors:** Hong Thai, James Lara, Xiaojun Xu, Kathryn Kitrinos, Anuj Gaggar, Henry Lik Yuen Chan, Guo-liang Xia, Lilia Ganova-Raeva, Yury Khudyakov

**Affiliations:** 1 Division of Viral Hepatitis, Centers for Disease Control and Prevention, 1600 Clifton Rd, Atlanta, GA 30329 USA; 2 Gilead Sciences Inc., 333 Lakeside Drive, Foster City, CA 94404 USA; 3 The Chinese University of Hong Kong, Hong Kong, China; 4 Present Address: Moores Cancer Center, University of California San Diego, La Jolla, CA 92037 USA; 5 Present Address: ViiV Healthcare, Research Triangle Park, NC 27709 USA

**Keywords:** Viral hepatitis, Genetic predisposition to disease

## Abstract

Tenofovir disoproxil fumarate (TDF) is one of the nucleotide analogs capable of inhibiting the reverse transcriptase (RT) activity of HIV and hepatitis B virus (HBV). There is no known HBV resistance to TDF. However, detectable variation in duration of HBV persistence in patients on TDF therapy suggests the existence of genetic mechanisms of on-drug persistence that reduce TDF efficacy for some HBV strains without affording actual resistance. Here, the whole genome of intra-host HBV variants (N = 1,288) was sequenced from patients with rapid (RR, N = 5) and slow response (SR, N = 5) to TDF. Association of HBV genomic and protein polymorphic sites to RR and SR was assessed using phylogenetic analysis and Bayesian network methods. We show that, in difference to resistance to nucleotide analogs, which is mainly associated with few specific mutations in RT, the HBV on-TDF persistence is defined by genetic variations across the entire HBV genome. Analysis of the inferred 3D-structures indicates no difference in affinity of TDF binding by RT encoded by intra-host HBV variants that rapidly decline or persist in presence of TDF. This finding suggests that effectiveness of TDF recognition and binding does not contribute significantly to on-drug persistence. Differences in patterns of genetic associations to TDF response between HBV genotypes B and C and lack of a single pattern of mutations among intra-host variants sensitive to TDF indicate a complex genetic encoding of the trait. We hypothesize that there are many genetic mechanisms of on-drug persistence, which are differentially available to HBV strains. These pervasive mechanisms are insufficient to prevent viral inhibition completely but may contribute significantly to robustness of actual resistance. On-drug persistence may reduce the overall effectiveness of therapy and should be considered for development of more potent drugs.

## Introduction

Resistance of viral strains to drugs is an important problem for patient management and public health. Viral drug resistance is usually associated with simple patterns of mutations involving only a few genomic sites^1–3^. One of the most studied and effective drugs are nucleotide and nucleoside analogs^4^. In hepatitis B virus (HBV), these analogs inhibit reverse transcriptase (RT) activity^5,6^. Development of HBV drug resistance is caused by specific viral mutations directly affecting recognition and binding of the analogs^7^ or excision of chain terminators by RT^8^, and may be accompanied by complementary mutations that correct fitness reduction usually associated with the primary mutation^9^. These patterns of mutations are generally referred to as a genetic barrier to resistance. Genetic patterns of greater complexity engender a greater genetic barrier to development of resistance^10^.

Although drug resistant mutations have a strong phenotypic effect, they are not independent from other genomic sites and genetic composition of the intra-host viral population. Estimates of the rates of mutation and viral replication indicate that all possible single and double mutations, and a large fraction of possible triple mutations are generated during each day of viral replication in infected hosts^11,12^, making many simple mutation patterns associated with drug resistance readily available to essentially any intra-host viral population. Nevertheless, despite such a wide presentation of drug-resistance mutations, not all viral strains develop resistance, indicating that phenotypic effects of these mutations are dependent on the genetic background to which they occur, emphasizing a significant role of epistasis and coevolution among viral genomic sites in development of resistance^13,14^. Therefore, strength of the genetic barrier is associated not only with complexity and availability of genetic changes required for resistance to viral population but with the overall fitness effects of these mutations in the background of the strain genetic composition^13^.

Worldwide, an estimated 248 million people had chronic HBV infection in 2015^15^. HBV is a small DNA virus, the replication of which involves an RT step^16^. Thus, drugs developed to control the human immunodeficiency virus (HIV) RT activity are also effective against HBV RT. Many of these drugs have a low HBV genetic barrier to resistance, resulting in frequent development of resistance^1,2^. Tenofovir disoproxil fumarate (TDF) is one of the nucleotide analogs effectively inhibiting HIV and HBV replication. Unlike many drugs, however, TDF is not associated with development of HBV drug resistance^17–20^. Nevertheless, TDF treatment results in a variable duration of HBV replication in different patients. Some patients experience a rapid HBV clearance, while others have detectable HBV after almost 2 years of TDF treatment^21,22^. While treatment response depends on a variety of host and viral factors, such differences could also be a result of genetic mechanisms that reduce TDF efficacy for some HBV strains without affording actual resistance.

Here, we show that differential responses to TDF are associated with genetic composition of the entire HBV genome, with RT playing a non-dominant role. The findings suggest the existence of various HBV genetic mechanisms contributing in different combinations to the rate of the HBV on-TDF persistence. Without causing resistance, the on-drug persistence mechanisms may mitigate effects of many HBV RT-inhibiting drugs, thus should be considered for the development of more potent therapies.

## Results

### Changes in intra-host HBV population

1,288 whole genome HBV sequences (954 unique sequences) were obtained from ten patients (P1–P10), with 114–171 (44–122 unique) intra-host sequence variants obtained from each patient. HBV sequences were obtained at three time-points (0, 4 and 40 weeks after initiation of TDF therapy) from all slow response (SR) patients. However, no sequences were obtained from specimens collected at week 40 from rapid response (RR) patients P5, P6 and P7, and only five and six sequences were obtained from RR patients P4 and P9, correspondingly.

**Figure 1:**
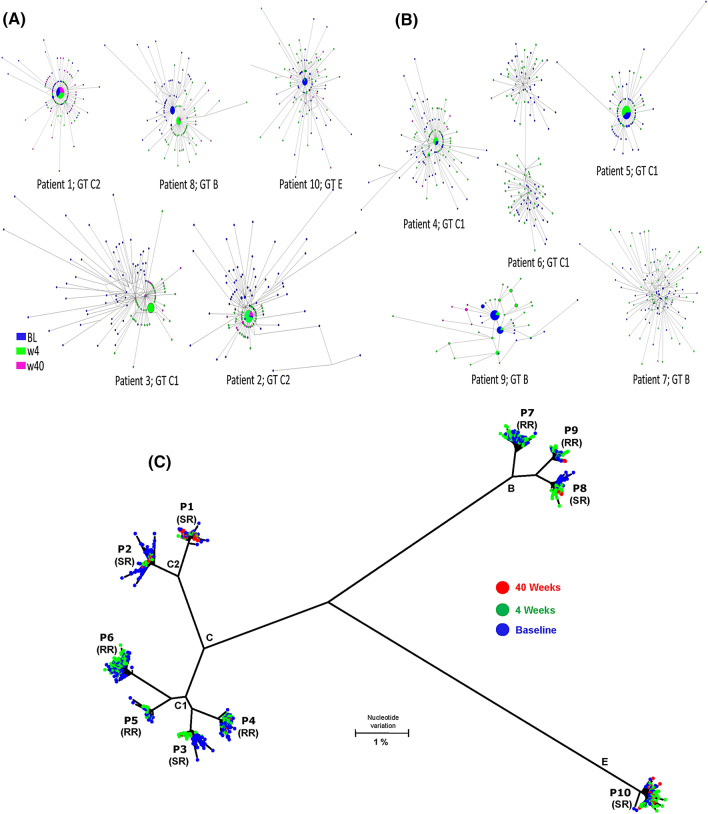
Heterogeneity and phylogeny of HBV quasispecies strains of genotype (GT) C, B and E. Shown are the median joining networks (MJN) of the full HBV genomic quasispecies sequences sampled from (**A**) five SR patients (P1-P3, P8 and P10) and from (**B**) five RR patients (P4-P7 and P9), and (**C**) phylogenetic tree. Sequences were sampled at three time points: baseline and week 4 and 40 during TDF therapy. GT C sequences organized into two clades or clusters (C1 and C2). Nodes in MJN and in phylogram tree represent HBV variants. Nodes colored based on time point of sampling (as denoted in color legend).

Among the RR cases, two (P6 and P7) were infected with a heterogeneous HBV population composed of many low-frequency variants at the baseline, which remained similarly diverse at week 4. However, three cases (P4, P5 and P9) were infected with HBV populations that had dominant or high-frequency variants. Although the dominant variants varied in frequency between time-points, they were present at week 4 (Fig. 1) but were replaced later with different variants in two cases, P4 and P9, who remained HBV PCR positive at week 40. Thus, in all RR cases, viral population largely preserved its structure at baseline and week 4, and experienced delayed shift, losing dominant variants, at week 40 in two cases, P4 and P9, who were still PCR-positive at the third time-point.

Among SR cases, two (P2 and P3) were infected with diverse HBV populations, containing only low-frequency variants at baseline, which became less heterogeneous at week 4 and 40. Both cases contained a high-frequency variant at week 4, which persisted throughout the observation to week 40 in P2 but was detectable only at week 4 in P3. The other three SR cases (P1, P8 and P10) had a high-frequency variant at baseline. In P1, a single variant was continuously dominant at all three time-points. In P8, the initial major variant was replaced with a different one at week 4, which remained detectable at week 40. In P10, the initial dominant variant declined in frequency by week 4 and turned undetectable at week 40 (Fig. 1).

Thus, while no substantial changes in the intra-host population occurred between weeks 0 and 4 among all RR cases, the intra-host HBV population experienced a detectable shift in three SR cases (P2, P3 and P8) between these two time-points. The rapid shifts in intra-host population of P2, P3 and P8, accompanied by increase in frequency of certain intra-host variants in P2 and P3 or by replacement of the dominant variant in P8, within 1 month after initiation of therapy indicate a capacity of these SR-HBV strains to adapt to TDF within a short period of time. This results in a slow HBV decline in patients on treatment, while none of the RR strains could produce intra-host variants of a similar replicative strength on TDF. Persistence of the major variant in P1 indicates that this SR strain was less sensitive to TDF initially, which, together with the observation of significant population shifts in the other three SR cases, suggests differential sensitivity of intra-host HBV variants to TDF, being especially detectable among SR strains.

### SR/RR-associated mutations

**Figure 2:**
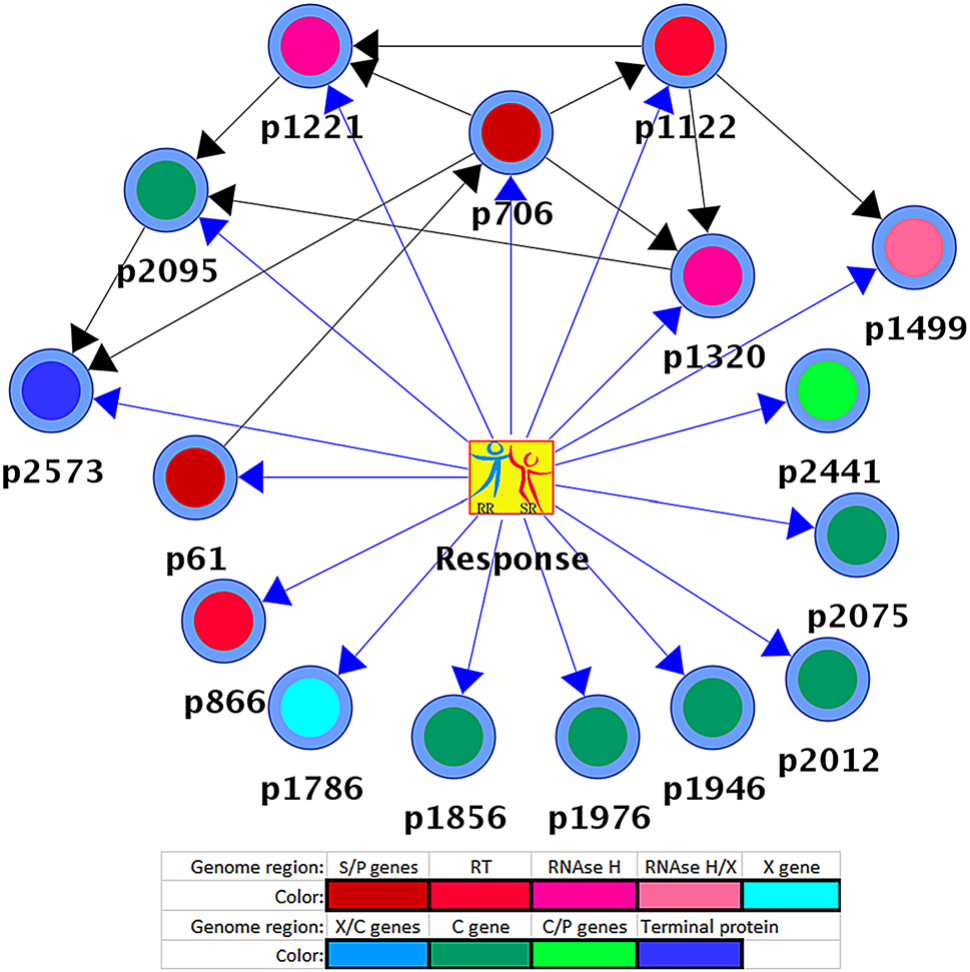
Relevant nt sites associated to the RR/SR. BN generated using full HBV genomic quasispecies sequences (N = 954) of GT C, B and E sampled from ten patients at three time points: baseline, and week 4 and 40 during TDF therapy. Round nodes in the graph represent 16 polymorphic nt sites (Table 1) and the square node represents the response (“target”) variable. Coloring of round nodes based on genomic region (see legend Fig. 4). Dependencies (relationships) between the response and nt sites are displayed as blue arcs and inter-dependencies between the sites as black arcs. The average strength of the relationship between a node and the target was small but significant (KL = 0.19, P < 0.05). However, four relationships in the network—arcs between the target and nodes representing genome positions (p): 866, 1946, 2075 and 2441—could not be statistically supported (P > 0.05). Nonetheless, this BN was found useful for prediction of RR/SR association (Tables S1 and Tables S2, in SI).

Considering significant effects of TDF on the structure of intra-host HBV populations, it is conceivable that HBV variants from the SR group may carry specific mutations affording a greater protection against TDF than mutations in variants from the RR group. Inspection of the nucleotide (nt) sequence alignment of all intra-host variants did not reveal any mutations completely specific to RR or SR. However, application of the correlation-based feature selection (CFS) algorithm^23^ allowed for identification of 16 nt sites associated with RR or SR classes (Table 1). These sites were found to be scattered across all HBV genes (C, X, P and S), with nine mutations affecting genomic regions encoding all four domains (terminal protein (TP), spacer (Sp), RT and RNAse H) of the P protein. Among the 16 sites, 5 are 3rd positions of codons of the P (N = 4) and C (N = 1) open reading frames (ORF) that are in genomic regions outside of the ORF overlap (Table 1). The association of the 16 nt sites with RR/SR was confirmed by the targeted analysis using naïve Bayesian Network (BN) (Fig. 2). BN analysis showed a significant association of polymorphism at site 2573 with RR/SR classes (Kullback–Leibler divergence (KL) = 0.78; *P* < 0.001). All intra-host HBV variants (N = 458) sampled from RR cases (P4, P5, P6, P7 and P9) had cytosine at this site, while intra-host variants (N = 393) from 4 SR cases (P1, P2, P8 and P10) had thymine at this site. Only SR P3 had intra-host HBV variants (N = 103) containing cytosine at site 2573. Moreover, the naïve BN was found to have accuracy of 99.7% (95% CI 99.4–100) in leave-one-out cross-validation tests, while achieving the expected accuracy (~ 50.0%) in randomly labeled data (Tables S1 and Tables S2 in SI).

**Table 1:** Polymorphic nt sites associated to RR/SR response in HBV strains of GT C, B and E.

Genome	Gene^a^	Protein domain (codon)^a^	Overlapping^a^
61	P 324	Sp 147 (1st)	S 143 (3rd)
706	P 539	RT 193 (1st)	S 358 (3rd)
886	P 599	RT 253 (1st)	–
1122	P 667	RT 331 (3rd)	–
1221	P 710	RNAse H 20 (3rd)	–
1320	P 743	RNAse H 53 (3rd)	–
1499	P 803	RNAse H 113 (2nd)	X 42 (3rd)
1786	X 413	X 138 (2nd)	–
1856	pre-C 43	pre-C 15 (1st)	–
1946	C 133	C 45 (1st)	–
1976	C 163	C 55 (1st)	–
2012	C 199	C 67 (1st)	–
2075	C 262	C 88 (1st)	–
2095	C 282	C 94 (3rd)	–
2441	P 45	Terminal protein 45 (3rd)	C 210 (1st)
2573	P 89	Terminal protein 89 (3rd)	–

Listed are the positions of the CFS-derived subset of 16 nt polymorphic sites found across several regions of the HBV genome that were strongly associated (Merit = 0.755) to the response rate characteristics of TDF-treated patients. The CFS algorithm^23^ was applied to the dataset of unique full-length HBV quasispecies sequences (N = 954) sampled from ten patients at three time points: baseline and week 4 and 40 during treatment. In total, 1,443 candidate subsets were evaluated by CFS (details in SI).

Position numbering is based on reference sequence: GenBank accession number AY233278.

^a^Gene, protein or protein domain abbreviations: polymerase gene (P), spacer (Sp), reverse transcriptase (RT), ribonuclease H (RNAse H), terminal protein, core gene (C), pre-core (pre-C), X gene (X), S gene (S). First, second or third codon positions are noted in parenthesis. Non-overlapping positions denoted by dash line “–“.

**Figure 3:**
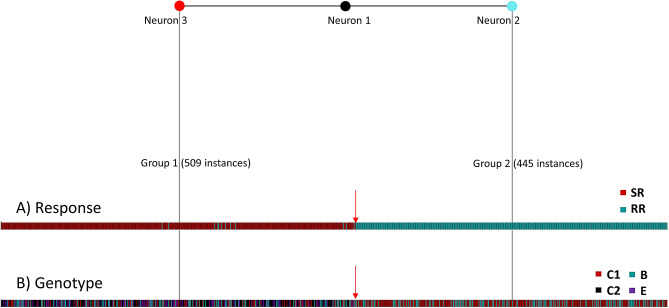
Physicochemical clustering of HBV sequences. Shown is the SOTA-based^24,25^ grouping, by (**A**) response and by (**B**) genotype, of physicochemical profiles representing full HBV quasispecies sequences (N = 954) collected from ten TDF-treated patients at three time points. Group 1 (neuron 3, in red) was mostly (97.4%) comprised of HBV GT C (clusters C1 and C2—see Fig. 1C), B and E strains sampled from SR patients. Group 2 (neuron 2, in cyan) was comprised of strains derived solely from RR HBV/C- & B-infected patients. Only thirteen out of the 47 unique variants sampled from one HBV/B-infected RR patient were found to be members of Group 1. Red arrow denotes boundary between the two groups. The 16 nt-based physicochemical profile representation of HBV sequences was generated using a scale of five physicochemical properties of DNA nt’s^26^.

Additionally, a self-organizing artificial neural network (ANN) model^24,25^ constructed using the 16 nt sites shown in Table 1 (see “SI Methods”) showed a clear partitioning of HBV variants into two clusters concordant with RR/SR (Fig. 3). Among HBV genotype C (HBV/C) and HBV genotype B (HBV/B), the model accurately identified 97.2% of the RR-associated sequences. Physicochemical profiles of the 16 nt sites from only 13 sequence variants of the RR HBV/B strain infecting P9 (six from baseline; six from week 4, and one from week 40) were similar to variants (N = 496) obtained from SR patients infected with HBV/B, HBV/C and HBV/E. Thus, although genetic analysis did not allow for identifying a single mutation clearly distinguishing RR and SR, a combination of several mutations scattered across the entire HBV genome were found to be strongly associated with RR/SR as a group, suggesting that on-drug persistence is complexly encoded in the HBV genome.

### The RR/SR association among HBV genotype C strains

**Figure 4:**
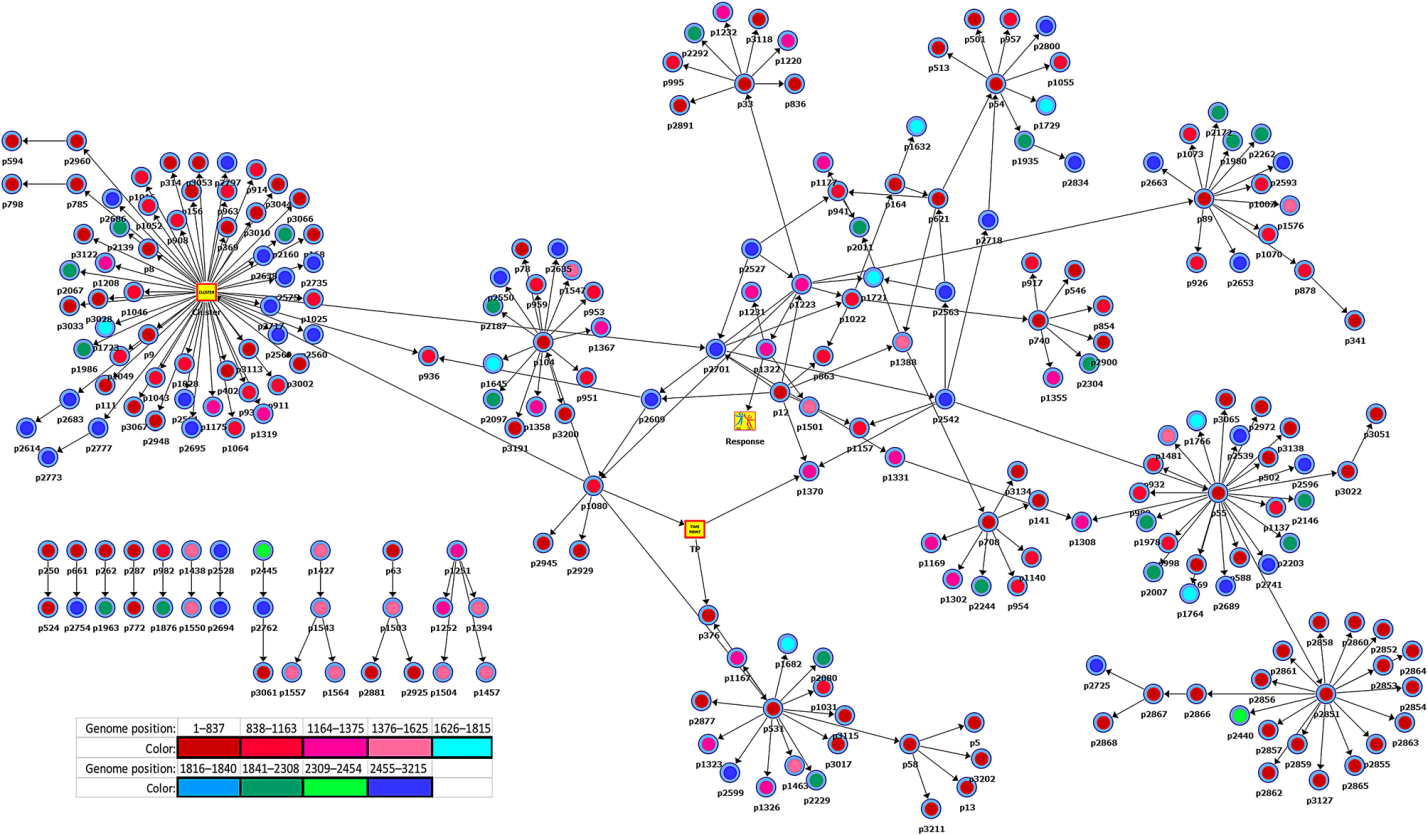
Genome-wide dependencies among polymorphic sites. BN generated using 799 whole-genome quasispecies from 6 HBV/C-infected patients treated with TDF (see “BN Section” in SI). Round nodes in the graph represent polymorphic nt sites and arcs represent significant (SC = 1.0, P < 0.001) dependency relationships. The BN comprises a major 215-varaible component (212 nt sites, and the response, time-point and phylogenetic cluster variables—square nodes in yellow) and 11 minor components representing 30 nt sites. Node coloring based on nine regions (genome positions in parenthesis): overlapping S–P genes (1–837), RT domain (838–1163), RNAse H domain (1164–1375), overlapping RNAse H–X (1376–1625), X gene (1626–1815), overlapping X–C genes (1816–1840), C gene (1841–2308), overlapping C–P genes (2309–2454) and Terminal protein domain (2455–3215). Position numbering based on reference sequence: GenBank accession number AY233278.

Considering a significant genetic diversity among HBV genotypes^27,28^, it is conceivable that molecular mechanisms responsible for the on-TDF persistence may be specific for each genotype. Thus, complex genetic encoding of the persistence suggested here may result from a set of simple genotype-specific genetic associations. Indeed, among the ten HBV strains used in this study, only six belonged to a single genotype C, while the other four belonged to genotypes B (N = 3) and E (N = 1). Since HBV/C strains were most represented, HBV/C sequences (N = 799) were used to generate BN (details in “SI methods”) to explore genotype-specific genetic associations with RR/SR. Among 1,020 HBV/C polymorphic genomic sites (~ 32% of all sites), 77.0% did not form significantly strong associations (SC ≥ 1; p ≤ 0.05) to each other (see Fig. S1 in SI). Meanwhile, ~ 21.0% of the sites were organized into a major BN component that included the RR/SR variable (Fig. 4), suggesting a certain genetic association of the involved sites with the on-TDF persistence. The BN was evaluated by Bayesian testing using Bayes factors (*Bf*)^29,30^ to measure the statistical significance of the influence of each state of every polymorphic site on the RR/SR state (see “SI methods”). Our analysis showed that nt states of polymorphic sites disconnected from the major component (N = 30) were non-informative with respect to the RR/SR state (exhibited neutral *Bf*; *Bf* = 0). However, *Bf* showed a strong association of 39.0% of nt states for 203 polymorphic sites composing the major BN component with the RR/SR as well as with phylogenetic clustering (see “SI methods”).

To sort out genetic associations with RR/SR vs. associations with phylogenetic clusters and time-points (Cluster and TP nodes in Fig. 4), we conducted target analysis (see “SI methods”). Among the 203 sites, 23 were found to be more significantly (KL ≥ 0.68; *P* < 0.*001*) associated with RR/SR than with phylogenetic cluster or time point (Fig. S2 in SI). The majority of these sites (N = 16) were located in three domains of the P protein: TP (N = 5), RT (N = 5) and RNAse H (N = 6). It is important that 4 of the 6 RNAse H sites (genome positions 1223, 1231, 1322 and 1501) were most strongly associated to RR/SR (Fig. S2B in SI). When the state for these sites is known, the state of the RR/SR variable becomes independent of the other variables in the BN (Table 2). Target analysis conducted without the 4 sites from RNAse H showed that the state of RR/SR variable can be accurately estimated from 72 polymorphic sites of the major BN component (Fig. 4). These sites had stronger associations (KL values ranging from 0.10 to 0.48) to RR/SR than to PC and T (Fig. S3 in SI). Considered together with *Bf* for each site in the BN (Fig. 4), these results suggest abundance of genetic pathways affecting RR or SR phenotypes, indicating involvement of many more genetic mechanisms in the on-drug persistence than usually associated with development of resistance. Although RNAse H seemingly plays a particularly important role in defining the rate of response to TDF for HBV/C strains, the identified 4 RNAse H sites had no association with RR/SR for HBV/B strains (data not shown), indicating differences in genetic mechanisms of on-drug persistence between these two genotypes.

**Table 2:** Frequency distributions of the RR/SR state given the observation of specific nt/aa states.

Genome nt positions^b^	nt states^a^	Response		aa positions^b^		aa states^a^	Response	
		SR (53.4%)	RR (46.6%)	P	RNAse H		SR (47.2%)	RR (52.8%)
1501	G (45.9%)	*98.8%*	*1.2%*	803^c^	113^c^	R (48.1%)	*98.0%*	*2.0%*
	A (54.1%)	*0.0%*	*100.0%*			H (51.9%)	*0.0%*	*100.0%*
1322	C (53.4%)	*100.0%*	*0.0%*	743	53	N (47.2%)	*100.0%*	*0.0%*
	A (46.6%)	*0.0%*	*100.0%*			K (52.4%)	*0.0%*	*100.0%*
						T (0.4%)	*0.0%*	*100.0%*
1231	A (53.7%)	*99.1%*	*0.9%*	713	23	R (51.9%)	*0.5%*	*99.5%*
	T (0.1%)	*100.0%*	*0.0%*			S (0.2%)	*0.0%*	*100.0%*
	G (46.2%)	*0.3%*	*99.7%*			Q (47.6%)	*98.0%*	*2.0%*
						L (0.2%)	*100.0%*	*0.0%*
1223	A (31.9%)	*100.0%*	*0.0%*	710^d^	20^d^	I (86.0%)	*38.8%*	*61.2%*
	G (21.5%)	*100.0%*	*0.0%*			M (13.7%)	*100.0%*	*0.0%*
	T (30.2%)	*0.0.0%*	*100.0%*			V (0.2%)	*0.0%*	*100.0%*
	C (16.4%)	*0.0%*	*100.0%*					

Frequency distribution of the SR/RR state associated to specific nt and aa polymorphisms (i.e., posterior conditional probabilities in BN) are shown in italiced cells.

Frequency distributions, in the data, of nt and aa states and of SR and RR phenotypes (i.e., prior conditional probabilities in BN) are shown in parenthesis.

BN analysis (details of analysis in SI) was performed on the dataset of unique full-length HBV GT C quasispecies nt sequences (N = 799) and polymerase aa sequences (N = 422) sampled from six patients (P1-P6) at three time points: baseline and week 4 and 40 during treatment.

^a^One-letter symbols denote the nt states: guanine (G), adenine (A), cytosine (C) and thymine (T); and the aa states: arginine (R), histidine (H), asparagine (N), lysine (K), threonine (T), serine (S), glutamine (Q), leucine (L), isoleucine (I), methionine (M) and valine (V).

^b^Position numbering in polymerase protein (P) and ribonuclease H (RNAse H) based on reference GenBank sequence AF458665.1.

^c^Four variants sampled at week 4 from RR patients (three from P5 and one from P4) had R at this position.

^d^All HBV quasispecies sequences from SR P1 and one variant sampled at week 4 from SR P3 had M at this position, and one variant sampled at baseline from RR P6 had V at this position.

### The RR/SR association of the HBV/C P protein

HBV drug resistance to nucleotide analogs is usually afforded by amino acid (aa) changes in RT of the P protein^1–3^. Although our analysis showed that nucleotide changes associated with RR/SR are distributed across the entire HBV genome, aa substitutions in P should be expected to play an important role in defining the rate of TDF response. Taking into consideration the aforementioned genotype specificity of the on-drug persistence, analysis was performed using P protein aa sequences from HBV/C only. It was found that 48 of 265 aa polymorphic sites from P form a major BN component (SC = 0.95; P < 0.001), with seven of these sites (positions in P: 307, 321, 624, 713, 743, 803 and 828) having a strong association (KL ≥ 0.41) with the RR/SR variable as determined by target analysis (Fig. S4 in SI). Importantly, the identified aa sites matching the nt sites showing a strong association with RR/SR (Table1 and Fig. S4 in SI).

Generally, nucleotide analogs inhibit the HBV RT activity. Our findings support an important role of RT in defining the HBV RR/SR phenotypes. To examine a potential contribution of the RT nt and aa variability to the rate of response to TDF among HBV/C strains, we conducted additional analyses. Prior to constructing a BN, all baseline sequences were initially mined and analyzed using the CFS algorithm (details in “SI methods”) to select a minimal subset of nt or aa polymorphic sites that maximize the conditional (posterior) probability of observing RR or SR. A minimum subset of 10 nt polymorphic sites (genome positions: 253, 280, 376, 458, 708, 828, 836, 926, 995 and 1006) as potential predictors (Merit = 0.527) of RR/SR was identified (Fig. S5 in SI). The nt polymorphic sites at position 836, 926 and 995 in RT, which were observed to be strongly associated (KL > 0.21; P < 0.001) with RR/SR in the BN (Fig. S3 in SI), were also selected by CFS. Although association of sites at position 253, 458 and 1006 to RR/SR was not statistically supported (*P* > 0.05), robust classification (CA = 100%) into RR or SR by a model using all these 10 selected nt sites (Fig. S5 in SI) suggests that the RT nt sequence contains information pertinent to the rate of response to TDF among HBV/C strains.

In addition, CFS analysis applied to the RT protein sequences (N = 247) sampled at baseline from the 6 HBV/C-infected patients identified a minimum subset of 22 polymorphic aa sites (Table S3 in SI), which were strongly associated (Merit = 0.511) as a group with RR/SR. BN constructed using these sites was shown to detect RR or SR with high accuracy (CA = 98.8%), while achieving the expected accuracy (CA = 53.4%) on random-labeled data. It is interesting that among the selected 22 sites, 6 located at the RT positions 82, 139, 153, 191, 223 and 233 have been reported as related to drug resistance^31–33^. Thus, the findings of groups rather than individual nt or aa sites strongly associated with RR/SR indicate that the differential sensitivity of HBV/C to inhibition by TDF is not defined by a single mutation and most probably involves either a single compound function or several simple functions of RT.

### Protein 3D-structure mapping

**Figure 5:**
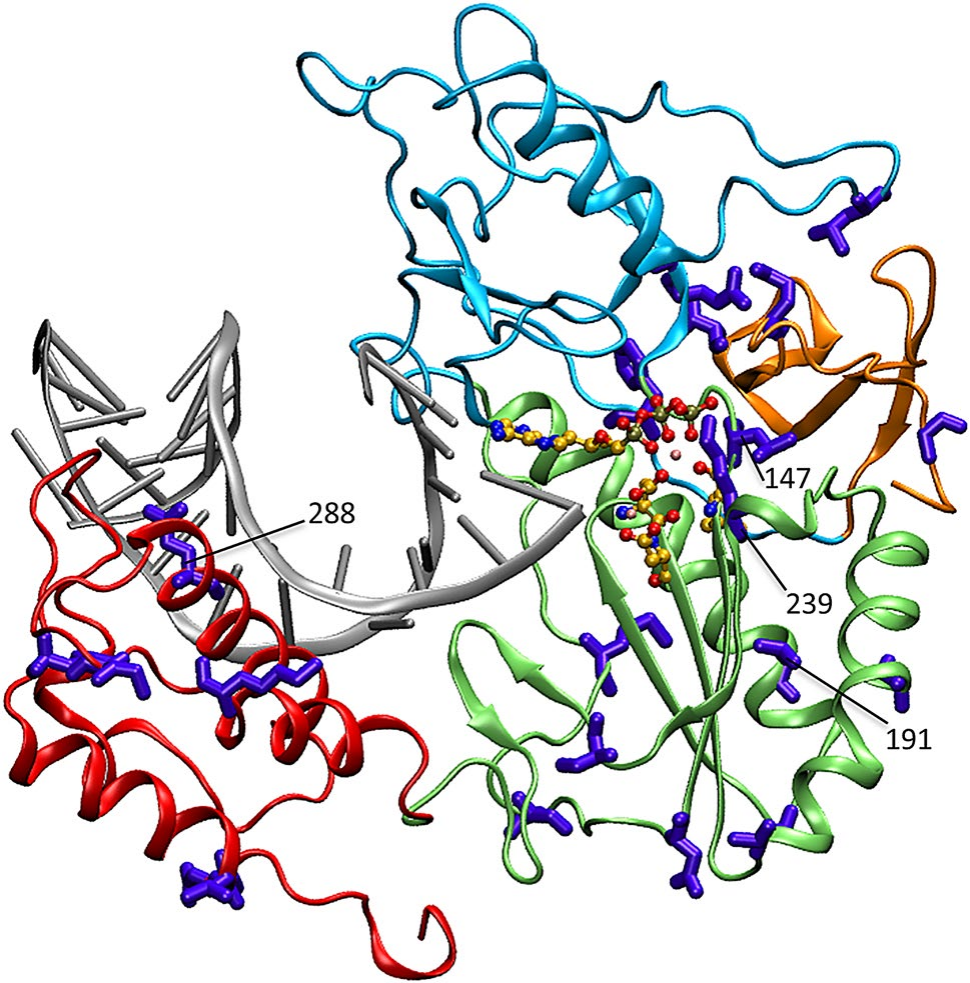
Mapping of HBV/C RT polymorphic aa sites relevant to SR/RR association. Shown is the predicted 3D structure of the HBV-RT/DNA-RNA/TFV-DP complex representative of the 345aa-long RT protein of HBV GT C strains^34^. The 22 aa sites represented in the RT-BNC (Table S3 in SI) are denoted as sticks (in purple). Potential effectors (N = 4) of the ligand–protein interaction are marked with corresponding RT positions. RT 3D-structure coloring scheme: fingers, in cyan and gold; palm, in green, and thumb, in red. TDF and DNA/RNA ligands are depicted with ball-and-stick (cpk colors) and cartoon (grey color) representations, respectively. Rendering was done using the VMD software^35^. Position numbering based on reference sequence: GenBank accession number AF458665.1.

The 22-selected polymorphic aa sites were mapped onto the 3D-structures of RT to identify structural effects of mutations at these sites that can potentially explain RR/SR phenotypes. Using the predicted HBV-RT/DNA-RNA/TFV-DP protein–ligand complexes^34^, the 3D-models were constructed for major HBV GT/C variants from 2 SR cases (P1 and P2) and an RR case (P5) (Fig. S6 in SI). Two aa sites, L147 and K239, were found to be in vicinity to the nt binding pocket (Fig. 5). Among all polymorphic aa sites in HBV/C RT, 2 other sites were identified that can potentially affect binding nt and DNA directly: V191, located in the alpha helix structure forming the nt binding pocket interface, and Q288, located in the alpha helix structure forming the DNA binding interface in the RT thumb domain. The states of these four sites were, however, identical among the three studied here HBV/C RT variants, indicating that genetic variation at these sites do not have a clear effect on the on-TDF persistence at least for the HBV variants from the three cases.

### Binding patterns of TDF to RT variants

To further investigate potential roles of the 22 aa polymorphic sites, analysis was conducted to characterize interaction between the diphosphorylated tenofovir (TFV-DP) and HBV RT. Analysis was performed using five predicted HBV-RT/DNA-RNA/TFV-DP protein–ligand complexes^34^ for the three aforementioned major HBV/C variants (from P1, P2 and P5) and two additional major variants from GT/B strains, one from RR patient (P9) and another from SR patient (P8) (Fig. S6 in SI). Analysis indicates that the TFV-DP binds near the YMDD motif (HBV RT active site; RT positions 203–206), with M204 and D205 contributing hydrophobic and negative-charge contacts, respectively (Fig. S7 in SI). The triphosphate end of TFV-DP is stably anchored to the binding pocket by hydrogen bonds (H-bonds) with Y148, T150, R110 and K149, and is strongly coupled by an Mg^2+^ chelation network. The detected residue-specific interactions and metal coordination coupling persist throughout MD simulations in all the predicted protein complexes of HBV/C and HBV/B (Table S4 in SI). The base-ring end of the TFV-DP is anchored by two persisting H-bonds with U-bases from the template RNA. Analysis also indicates that sites L147 and K239, mapped in vicinity of the binding pocket (Fig. 5), contribute to the TFV-DP binding interaction, providing, respectively, hydrophobic and positive-charge contacts (Fig. S7 in SI). Thus, the data, summarized in Table S4 (in SI), show no substantial differences in TFV-DP-binding patterns among the studied HBV RT variants, suggesting that the TDF binding to RT is not strongly associated with RR/SR.

## Discussion

Genetic analyses conducted here indicate that the differential response to TDF among HBV strains is not associated with a specific mutation, a unique mutation pattern or a single HBV protein. Rather, capacity to the on-TDF persistence is a complex genetic trait, which is intricately encoded across the entire HBV genome. Identification of 16 sites from different HBV genomic regions (Table 1), which are strongly associated as a group with the TDF response, suggests the existence of a few compound or many simple genetic pathways contributing to the TDF response. These sites are distributed across all HBV genes, with nine sites located in genomic regions encoding all four structural domains of the P protein. Only three sites, one of which is synonymous, were found to be in the RT domain, indicating that RT does not play a major role in defining HBV persistence on TDF. Thus, there is an important difference between molecular mechanisms responsible for resistance to nucleotide and nucleoside analogs and for on-TDF persistence. While functional dominance of RT mutations in development of resistance is well established^31–33^, contribution of RT to controlling the level of HBV replication during TDF treatment, although essential, is seemingly limited. This conclusion is supported by the lack of difference in TDF binding by RT from persistent and rapidly declining HBV variants in the 3D-models implemented here. Thus, a potential involvement of all HBV proteins and limited contribution of RT to the protracted HBV replication on TDF suggest that genetic mechanisms of on-drug persistence and drug resistance are essentially different.

Molecular mechanisms of resistance to nucleotide and nucleoside analogs generally lead to a significantly reduced drug recognition and/or binding by the RT active center^7^ or excision of chain terminators^8^, which result in indefinite survival of the resistant HBV strain in presence of drug. Usually, simple mutation patterns are associated with resistance^1–3^. Thus, small genetic changes result in a very strong phenotypic effect, making specific drug resistance readily selectable for many viral strains. In contrast, protection afforded by on-TDF persistence is incomplete. It only slows the decline of HBV population during treatment. A lesser phenotypic effect associated with complex mutation patterns seemingly makes on-drug persistence less selectable during TDF treatment. However, phylogenetic analysis showed rapid genetic changes in intra-host HBV populations of 3 SR strains (P2, P3 and P8) and continuous presentation of a dominant HBV variant in P1 during treatment, indicating a degree of adaptation of HBV population to TDF and certain resilience of some variants on treatment. The structure of intra-host HBV populations of RR strains, though, does not change as rapidly, showing inability to adapt to TDF. These observations indicate that not only do HBV strains differ in their ability to persist, there is also a substantial difference among intra-host HBV variants in their capacity to replicate on TDF. Thus, even small genetic changes generally observed among closely related intra-host HBV variants of a single strain seem to contribute to variation in sensitivity to TDF. Although the genetic mechanisms of on-TDF persistence can be disabled or enabled by few mutations in SR strains, these mechanisms cannot become fully functional in RR strains despite experiencing large numbers of mutations as in patients P6 and P7 infected with highly diverse HBV populations, which indicates a non-uniform distribution of the trait in the HBV genetic space.

A complex genetic encoding among SR strains coupled with small adaptive changes among intra-host HBV variants specific for each SR strain suggests the existence of many simple genetic mechanisms, various combinations of which set a specific path to persistence in each SR strain. In addition, it argues against the existence of a single or a dominant mechanism across all HBV strains, as generally observed for the actual drug resistance associated with simple and specific mutation patterns for all resistant strains. This observation suggests that the exact genetic mechanisms responsible for on-drug persistence may vary among HBV strains. The identification of different mutation spectra associated with persistence between HBV genotypes B and C, and lack of a single mutation pattern among persistent HBV strains studied here lends support to this supposition.

Owing to a high mutation rate and a large intra-host population size, it is estimated that HBV experiences all possible single and double mutations every day of infection in each infected individual^13^. However, despite the continuous occurrence of drug-resistance mutations, not all HBV strains develop resistance, indicating a fundamental role of HBV genetic background in phenotypic presentation of these mutations^13^. Epistatic connectivity among HBV sites is dense and can be organized into a network^36^. Genetic analyses show that this network defines HBV predisposition to drug resistance, making resistance mutations functionally acceptable in some HBV strains and, thus, selectable during treatment^14^. Like the resistance mutations, functional presentation of the TDF adaptive mutations in SR strains is epistatic or depends on the genetic background to which these mutations occur. Differences in genetic predisposition to persistence between SR and RR strains may explain adaptation of SR strains and lack of adaptation of RR strains to TDF treatment. Drug resistance and persistence are either convergent or independent of ancestry^14^, but persistence is highly genetically abundant or controlled by many genetic mechanisms, which alone are not as robust as resistance in controlling response to drugs and just protract HBV replication.

Cross-resistance of HBV strains to different nucleotide and nucleoside analogs is common^1^. Response to TDF, however, does not involve the development of actual resistance. Nevertheless, HBV infected patients preliminary treated with lamivudine or adefovir may have delayed or attenuated responses to TDF^18,37, 38^, suggesting a cross-selection for on-TDF persistence resulted from existence of genetic pathways for persistence shared by these three drugs. Genetic mechanisms of on-drug persistence may operate along with mechanisms of resistance. However, their effect is likely masked by the phenotypically dominant resistance. Identification of an HBV genotype A/G recombinant strain surviving during lamivudine treatment without development of the well-known lamivudine-resistance mutations^14^ suggests the existence of molecular mechanisms of on-lamivudine persistence, which are different from the actual lamivudine resistance.

The mechanisms of on-drug persistence are likely genotype specific. Indeed, a delayed response to TDF was observed for HBV genotype G^38^. Variation in susceptibility to TDF was reported for HBV genotype A vs. genotype C^39,40^. Here, mutation patterns associated with RR/SR were found to be different between HBV strains from genotypes B and C, additionally supporting the existence of genotype specific genetic pathways contributing to the TDF response. The nature of these mechanisms cannot be identified from the patterns alone. However, a strong association of the RNAse H sites with RR/SR in genotype C suggests a role of the enzymatic activity in on-TDF persistence. Although not yet observed for HBV, in HIV, mutations affecting the RNAse H conformation facilitate resistance to RT inhibitors likely by slowing degradation of the RNA genome during viral replication and, thus, providing more time for dissociation of the drug from the inhibited RT^41^. It is important to note that this mechanism is not specific to a certain drug. Many mechanisms associated with T- and B-cell responses^42–44^, as well as with functional states of the basal core promoter and pre-core regions of the HBV genome^45^ somewhat nonspecifically contribute to susceptibility to drugs and, thus, may serve to promote HBV persistence in absence of actual resistance.

In conclusion, capacity of HBV strains to persist on TDF is a complex trait genetically associated with mutations at many sites of the HBV genome. However, small genetic variations distinguishing persisting from non-persisting intra-host HBV variants indicate a potentially simple genetic nature of on-TDF persistence in each SR strain; while inconsistent presentation of these mutations among SR strains indicates a specific nature of these simple genetic mechanisms operating in each case. In contrast to drug resistance which is encoded by a dominant genetic mechanism across HBV strains, on-TDF persistence is likely controlled by many genetic mechanisms, each of which differentially operates in every persistent HBV strain. Although incapable to offset completely inhibition by drugs, on-drug persistence may contribute to or modify overall resistance. With drugs becoming ever more efficient, it is conceivable that complete resistance may become uncommon, and clinical management will rather face diminished responses to drugs, making mitigation of on-drug persistence essential for improving further quality of patients’ care by reducing duration of treatment as well as its cost. Understanding of genetic mechanisms of on-drug persistence should help in devising more potent drug therapies.

## Methods

### Patients

Whole-genome HBV quasispecies from ten immune tolerant patients (identified from Study GS-US-203-0101^46^) were used for this study. All patients provided written informed consent. All methods were carried out in accordance with relevant guidelines and regulations. The study was approved by the Institutional Review Boards of each participating institution (Centers for Disease Control and Prevention’s Institutional Review Boards). All patients had received TDF monotherapy, and were matched by HBV titer, ALT and HBeAg at baseline. Patients were evaluated at base line, week 4 and week 40. Five patients had a slow response (SR), never achieving HBV DNA < 400 copies/ml by week 96, while five had a rapid response (RR), achieving HBV DNA < 400 copies/ml by week 96. Demographics and clinical features of patients are presented in the SI, Table S5.

### Nucleic acid extraction and HBV whole genome quasispecies sequencing

Total nucleic acid was isolated from serum samples using the robotic Roche MagNA Pure LC system (software version 3.0.11) and the MagNA Pure LC Total Nucleic acid isolation kit (Roche Diagnostics GmbH, Mannheim, Germany), and eluted in 50 μl of lysis buffer according to the manufacturer’s instructions. Nearly full-length genomes of HBV genotype B (GT/B) and genotype C (GT/C) strains were amplified using two rounds of PCR as previously described^47^. Further details can be found in “SI Methods”.

### Median joining network (MJN)

Intra-host heterogeneity of HBV strains at baseline, week 4 and week 40 was evaluated by MJN, constructed using the Phylogenetic Network software (NETWORK, version 4.112, Fluxus Technology Ltd, Suffolk, England^48^ (https://www.fluxus-engineering.com/sharenet.htm).

### Bayesian networks (BN)

Genome-wide site-specific dependencies among nt/aa polymorphic sites in HBV genomes was assessed by BN modeling^49^. BN of polymorphic sites were estimated from alignments of HBV sequences using the SopLEQ method^50,51^ and a structural coefficient (SC) influence = 1 (significance threshold). The sequence alignment was done using the CLUSTALW program embedded in MEGA^52^ (version 6.06 https://www.megasoftware.net/). Prior to BN analysis, each sequence in the alignment was associated with respective metadata corresponding to response rate, genotype/subtype and time point of sampling. BN analyses were performed in three steps: learning step (SopLEQ method), analysis of associations and inference in order to characterize associations with response rates to TDF (target node). The Kullback–Leibler (KL) divergence^53^ was used to measure the strength of a direct relationship (link) between two variables (nodes), and the Bayes Factor (BF) metric^29,30^ to measure the impact of any given state of a node on the observed state of the target node. BN analyses were done with the BayesiaLab software (BayesiaLab, version 5.0.2 PE, Bayesia SAS, Laval, France, (https://www.bayesialab.com). Additional details are in “SI Methods”.

### Feature selection (FS)

Statistical^54^ or machine-learning^55^ FS methods generally provide efficient means for identifying the most useful attributes for classification or regression tasks and are commonly employed to reduce the dimensionality of the data (i.e., number of attributes) without negatively affecting the accuracy of the prediction. FS was applied to HBV full or partial genome and protein sequence data, which comprised unique quasispecies variants within a sampled host. Samples were obtained from HBV-infected patients: six infected by HBV genotype C (HBV/C), three by genotype B (HBV/B) and one by genotype E (HBV/E). As in the case of BN analyses, FS analysis was performed on the genomic and/or protein sequence CSV formatted data, where each sequence variant was respectively annotated with corresponding RR or SR class-labels.

The correlation-based feature selection (CFS) algorithm was used for FS analysis of the sequence data, which is an FS technique based on the Merit heuristic^56^. Merit-based heuristics are founded on the idea that good feature subsets contain features highly correlated with the class label, yet poorly inter-correlated with each other. The basic strategy of this merit-based heuristics is to find the best minimal subset of features associated to a class label by accounting for the class-feature correlation and feature-feature correlations. In other words, CFS takes into account the usefulness of a feature subset for predicting the class label, while accounting for the level of inter-correlation between the features within the subset. Here, polymorphic sites comprised in a feature subset were evaluated by CFS to measure their joint (combined) correlation with respect to the class-label and inter-correlation among themselves. Merit measures returned by the CFS evaluation were then used to select the best feature subset for prediction of the HBV variants RR or SR association. We used the feature subset evaluation function implemented in WEKA (version 3.17, https://www.cs.waikato.ac.nz/ml/weka/)^57^, which is formalized as follows,

$$Merit_{S} = \frac{{k \cdot \overline{{r_{{cf}} }} }}{{\sqrt {k + (k - 1) \cdot \overline{{r_{{ff}} }} } }}$$

where, $\overline{{r_{{cf}} }}$ is the average (avg.) class-feature correlation, and $\overline{{r_{{ff}} }}$ is the avg. feature-feature intercorrelation in the feature subset *S* containing *k* features.

CFS identified among many feature subsets the most useful RR/SR predictive feature subset in HBV quasispecies genomes and proteomes. Polymorphic sites comprised in the best feature subset were then used to build the classification models presented herein. We note that other feature subsets may also have strong RR/SR predictive usefulness. However, due to statistical^58^ and computational limitations^59,60^ associated with FS methods, including the limitation in our patients cohort size (N = 10), it is not possible to determine the ground truth about which features are causal (or relevant) factors for the RR/SR characteristic. Nevertheless, we used the classification theory approach to examine the degree of reliability by which nucleotide or amino acid variations at the polymorphic sites identified by CFS help to associate HBV variants to the host’s RR/SR characteristics, and to establish the unlikelihood that such association could be attributed to genotype- and/or patient-related biases (refer to SI: Tables S1, and Table S2 & Fig. S5, respectively) or to random statistical correlations (details in “SI Methods”). Although the CFS-selected features identified herein as biomarkers for association of HBV strains to RR/SR are not definitive, and genomics experimentation is needed, the classification theory approach strongly supported the importance of CFS-selected features as biological factors that contributed to the specific and accurate identification of the HBV RR/SR predisposition to TDF treatment.

### Classification models

Classifiers to identify/detect RR- and SR-strains were constructed using supervised and unsupervised machine learning^61^. Classification accuracy (CA) performances of supervised BN was evaluated using WEKA’s Experimenter module (version 3.6.1^57^, https://www.cs.waikato.ac.nz/ml/weka/). An unsupervised self-organizing ANN was built using the self-organizing tree algorithm (SOTA)^24,25^ embedded in the KNIME software (KNIME Analytics Platform for Linux, version 2.8.2, Knime AG, Zurich, Switzerland, 2013^62^ https://www.knime.com/). Further details can be found in “SI Methods”.

### RT 3D-structure modeling

Structural modeling of five representative SR/RR-associated RT protein variants and MD simulation experiments was performed as previously described^34^. Ligand–protein interaction graphs were generated using the Maestro software (Schrödinger Release 2014-1: Maestro, version 9.7, Schrödinger, LLC, New York, NY, 2014). The 3-dimesional (3D) rendering of protein structures was done using VMD software^35^ (version 1.9.2; https://www.ks.uiuc.edu/Research/vmd/). Further details can be found in “SI Methods”.

## Supplementary information

Supplementary Information.

## Data Availability

The sequence data that support the findings of this study were submitted to GenBank (accession numbers: MT426248–MT427201) and will be available to the public once they are processed. HBV data and datasets are available from the corresponding author upon reasonable request.
