# Supplementary material for: Complex genetic encoding of the hepatitis B virus on-drug persistence
Source: Sci Rep. 2020 Sep 23;10:15574. doi: 10.1038/s41598-020-72467-9 (PMC7511938; doi:10.1038/s41598-020-72467-9)
Supplement: Supplementary file 1 — Supplementary Information. [file 41598_2020_72467_MOESM1_ESM.docx]

**Complex genetic encoding of the hepatitis B virus on-drug persistence**

Hong Thai^1§*^, James Lara^1§^, Xiaojun Xu ^1¶^, Kathryn Kitrinos^2¶¶^, Anuj Gaggar^2^, Henry Lik Yuen Chan^3^, Guo-liang Xia^1^, Lilia Ganova-Raeva^1^ and Yury Khudyakov^1^

^1^Division of Viral Hepatitis, Centers for Disease Control and Prevention, 1600 Clifton Rd, Atlanta, GA 30329

^2^Gilead Sciences Inc., 333 Lakeside Drive, Foster City, CA 94404.

^3^The Chinese University of Hongkong, Hongkong.

^§^**H.T.** and **J.L.** equally contributed to this work.

^¶^Current address: Moores Cancer Center, University of California San Diego, La Jolla, CA 92037

^¶¶^Current address: ViiV Healthcare, Research Triangle Park, NC 27599

^*^To whom correspondence should be addressed. Email: guy8@cdc.gov

**SI Methods**

1. **Bayesian network (BN) learning**

A BN is a probabilistic graphical model, where nodes in the graph represent random variables and directed arcs between the nodes represent relationships ^1,2^ . Directed arcs define parenthood ordering among variables and encode the probability distributions in data. Given a finite set $S=\left\{ X_{i},\cdots,X_{n} \right\}$ of random variables, where $X_{i}$ can take any value in $S$, a BN is a directed acyclic graph (DAG), $G=\left\{ V,E \right\}$, that encodes the joint probability distribution over $S$, where $V$ are nodes in $G$ corresponding to random variables $\left\{ X_{i},\cdots,X_{n} \right\}$ and $E$ are edges in $G$ represent direct dependencies between the variables. Each node $X_{i}$ is associated with a conditional probability distribution (CPD), $P\left( X_{i} | P_{a}\left( X_{i} \right) \right)$, that quantifies the effect of the parents on the node, where $P_{a}\left( X_{i} \right)$ denotes the parents of $X_{i}$ in $G$. The pair $\left( G,CPD \right)$ encodes the joint probability distribution $P\left( X_{i},\cdots,X_{n} \right)$ given $G$. The joint probability distribution over $S$ from $G$ is factorized as:

$$P\left( X_{i},\cdots,X_{n} \right)=\prod_{i} P\left( X_{i} | P_{a}\left( X_{i} \right) \right)$$

Here, HBV genomic and/or proteomic quasispecies sequence data was used to learn (or infer) BN DAG (i.e., $G=\left\{ V,E \right\}$), where nodes in *G* represent polymorphic nucleotide (nt) or amino acid (aa) sites $\left\{ X_{i},\cdots,X_{n} \right\}$ and the CPD associated to a node encode the prior distribution of observed residue states in $X_{i}$. Three additional variables (target varaibles) representing response (states: RR and SR), time point of sampling (states: baseline, weeks 4 and 40) and phylogenetic clusters (states: clusters C1 and C2) were included in BN. For BN learning and analysis, the genomic and/or protein sequence alignments in FASTA format were exported to CSV format (comma delimited data) and each sequence variant was respectively annotated with the class label RR or SR in accordance to the observed response of its corresponding host, including time point of sampling and phylogenetic cluster annotations. Three BN models were constructed (learned) from the HBV genotype C data) to represent the probabilistic relationships among polymorphic sites and the three target variables: BN of full-length genomes, BN of pol proteomes and BN of RT proteomes.

Because BN provides a complete model of probabilistic distributions for variables and their relationships, BN models can be used to answer probabilistic queries about the state of a subset of target variables when other variables are observed (evidence features). The process of computing the posterior distribution of variables is achieved in BN by computing marginal probabilities for each target node given information on the states of a set of observed nodes, a process known as probabilistic inference. In the absence of any observations, this computation is based on a priori probabilities and, when observations are given, the information is integrated into BN and all probabilities are updated accordingly. Additionally, unsupervised techniques are available that can be used for automatically learning BN *G* from data and carrying out probabilistic inference.

Given that learning a BN from data has been proven to be NP-hard ^3^ and that our sample size is relatively small, a heuristic score-and-search-based approach was used to learn the BN from HBV quasispecies data. In general, such approaches have two components: a scoring function, used to evaluate how well the learned BN fits the data, and a search strategy, which consists of a learning algorithm to identify BN structure(s) with high scores among the possible structures in BN space ^1,2^. Here, the Minimum description length (MDL) algorithm ^4,5^ was used as the scoring function. The MDL score is a criterion based on information theory that favors a BN that provides the shortest description of the data. The MDL score has been shown to have better performance than other scoring methods in BN structure learning tasks ^4^. Also, this score is conservative and returns by default highly significant relationships. Given *BN = (G, CPD)*, and a training dataset *D*, the MDL score of BN is defined as,

$${MDL}_{Score}\left( BN | D \right)=MDL\left( BN \right)+MDL\left( D | BN \right)$$

where the first term, $MDL\left( BN \right)$, is the description length of the BN (number of bits required to encode BN parameters – structural complexity) and the second term, $MDL\left( D | BN \right)$, is the negative log likelihood of BN model given $D$ (gives the number of bits necessary to describe $D$ with BN). The structural complexity (SC) coefficient was preset prior to the start of BN learning. Unless otherwise specified, the SC coefficient was set equal to 1.0.

As a search strategy for identifying the best BN structure (i.e., BN with high MDL scores), we used an unsupervised learning algorithm based on the EQ method ^6^. This method, which is based on searching the equivalent BN classes (i.e., $G$ representing the same conditional dependencies), has been shown to be efficient for finding the optimal BN model^[[1]](#footnote-1)^ of the data ^6,7^. The best BN models were then selected for BN analyses. Inference/construction of BN, and analyses described in the Section below, were conducted using the BayesiaLaB™ software version 5.0 (Bayesia SAS, Laval, France).

1. **BN analyses**

Relationships among HBV nt/aa sites and the therapy outcome RR/SR were examined using probabilistic graphical models of BN, where nodes in the graph represent nt or amino acid (aa) sites, time point of sampling, phylogenetic cluster and therapy outcome) and links between the nodes represent relationship. Unlike the undirected independence graphs, BN DAGs provide a more complex notion of the relationships. This includes the notion of the conditional probability and directionality of the relationship. Links connecting two variables (nodes) in the graph are represented as arcs, which may project toward the node (incoming links) or from the node (outgoing links), thus specifying the direction of influences among variables. Relationships between variables in a BN may be interpreted as causal (22). The CPD in BN are represented in the conditional probability tables (CPTs) of the variables in the network. Here, CPTs encode probability distributions of nt/aa states at each polymorphic site and probability distributions of RR/SR states associated to nt/aa states of polymorphic sites.

1. **Relationship (dependency) Analysis**

The strength of dependencies among interrelated variables (i.e., nodes in $G$ linked by an arc) was inferred by computing the Kullback-Leibler distance (KL-distance) ^8^, also known as KL-divergence. Say $A\to B$ in $G$, then KL-divergence between CPTs of directly linked variables $\left( A,B \right)$ was measured with and without the arc. KL-divergence, $D_{KL}$, allows for comparing two probability distributions, $P$ and $Q$,

$$D_{KL}\left( P\left( X \right) || Q\left( X \right) \right)=\sum_{X} P\left( X \right){log}_{2}\frac{P\left( X \right)}{Q\left( X \right)}$$

where $P$ is the BN with the link and $Q$ is the BN without the link.

This is a statistical measure of dependency between variables; the greater the KL-divergence is, the greater the strength of the relationship.

KL-divergence was also used to identify the most important variables in learned BN models. Identification of such variables was based on the assumption that important variables are more likely to have greater numbers of strong relationships than variables who are less influential or relevant. The importance of a variable was defined in terms of a node’s strength in BN, which was estimated from the KL-divergence value of arc(s) associated to a node.

In addition, Pearson’s chi-square $\left( X^{2} \right)$ test for independence was computed from BN using the KL-divergence. The *p* values reported herein represent independence probabilities of $X^{2}$ tests for each relationship. Mutual information (MI) between directly linked variables was computed for each arc of the BN to determine the amount of information contributed by each node to the target node. The MI between two variables $\left( X,Y \right)$ connected by an arc in the BN is defined as,

$$I\left( X,Y \right)=\sum_{x\in X} \sum_{y\in Y} p\left( x,y \right){log}_{2}\frac{p\left( x.y \right)}{p\left( x \right)p\left( y \right)}$$

1. **Target analysis**

Target analysis was performed to infer which variables are more important to the knowledge of any observations (states) taken by the target variable. Nodes encoding the RR/SR response, BL/4w/40w time points of sampling and C1/C2 clusters were specified as the target variable. Here, analysis was focused on the response target variable using the remaining variables in BN to observe effects on frequency distributions of nt/aa states.

1. **Classification models**

Two computational classification methods were used in this study: Bayesian network and artificial neural network classification methods. Classification models were constructed using the CFS-based feature subsets and evaluated on the CSV formatted and SR/RR-annotated sequence data comprising unique HBV QS sampled from all 10 HBV-infected (N=954).

1. **Bayesian network classification (BNC) method**: is a Bayes’ theorem-based probabilistic graphical method where nodes in the graph (*G*) represent random variables and directed vertices (arcs) in *G* encode probability distributions in data ^9^. Bayes’ theorem-based methods have been shown to deliver accurate results when specific manifestations**^[[2]](#footnote-2)^** in data have high specificity ^10^.

This method was applied to generate a BN classifier and evaluate a subset of 16 HBV features. Briefly, automated learning of BN consists of two stages: 1^st^ stage: structure learning (herein, based on the K2 algorithm ^11^ and 2^nd^ stage: posterior probability estimation learning (herein, based on k2 priors). Further details about BN learning processes are fully discussed in Korb & Nicholson (2004). Construction of the BNC graph (*G*) was initiated first as a naïve BN (i.e., only arcs from the class node pointing towards variable nodes in *G*), using a SC threshold greater or equal to 0.70. Then, interdependencies among the variable nodes (represented in *G*, as arcs between variable nodes) were automatically derived (i.e., learned) from the HBV sequence quasispecies dataset (N=954, all data; or N=799, HBV/C). The number of variable nodes in the *G* corresponded to the size of the CFS-based feature subset (N=16). Classification performance tests were then conducted on the learned BNC.

Classification performance evaluation of learned BN was performed during the training and testing phases and involved calculating the class probability, $p(c)$, of QS instances given their input features. The ultimate goal of our system was to find an optimal classification for each instance (i.e., the true class-label, ***C_i_***, of an HBV variant). In BNC, the arguments of the maxima (argmax) function is useful for finding the ***C_i_*** that an instance and features are associated with. To do this, argmax computes probabilities for each ***C_i_*** and selects the ***C_i_*** that maximizes this probability, which can be formulated as follows,

$$argmax c of p(k_{1}|c)*p(k_{2}|c)*\cdots*p(k_{n}|c)*p(c)$$

where, $p(k_{n}|c)$ is the probability of observing a feature $k_{n}$ given the class *c*.

**1(a). BNC classification performance evaluation**: The learned BNC was evaluated by 10-fold-cross-validation (10x-CV), where the BNC output (decision) boundary between the RR and SR classes was set at a threshold of 0.5 (i.e., ≥ 0.5 = RR and <0.5 = SR). The HBV quasispecies sequence data (assigned with the associated observed labels, OL) was randomly divided into 10 subsets of roughly equal size without grouping instances by patient. The same partitioning was applied to data where HBV quasispecies sequences were randomly labeled (RL) as RR or SR classes. The learned BNC was trained on nine data subsets and then tested on the respective held-out testset. This was repeated until all 10 data subsets in each OL and RL datasets were tested. We note that the held-out data subset used to test the classification performance of the learned BNC trained on nine RL subsets was re-assigned the correct observed classes prior to testing. The RL dataset served to examine for the potential presence of random statistical correlations in the data. If variables (or selected features) represented in the BNC do not have spurious correlations to the RR/SR classes, then classification performance on the held-out data subset is anticipated to deteriorate to the expected classification accuracy of 50%.

We also conducted experiments where instances were grouped by patient and by HBV genotype. In leave-one-patient-out cross-validation (LOOCV-P) experiments, the sequence data from patients was divided into two data subsets: a training dataset, consisting of HBV sequences sampled from a group of patients and a test dataset, comprising sequences from the left-out *i*th patient. The learned BNC was trained by 10x-CV on the trainset and then tested on the respective testset of the left-out patient. Training/testing cycles were repeated until all train/test pairs were evaluated. The same procedure was applied to randomly class-labeled datasets. Briefly, five randomly class-labeled datasets were generated from each training dataset and used to train the learned BNC by 10x-CV, which was then tested on the corresponding, non-randomly labeled, testset pair (i.e., sequence data from held out patient).

In leave-one-genotype-out (LOOCV-GT) experiments, the quasispecies sequence data (N=954; 10 patients) was divided into two GT-specific data subsets^[[3]](#footnote-3)^; one dataset consisting of QS sequences sampled from HBV GT C-infected patients (N=611; 6 patients) and another comprising QS sequences sampled from HBV GT B- and GT E-infected patients (N=343; 4 patients). The learned BNC was trained one data subset by 10x-CV and then tested with left-out data subset. The training/testing cycle was performed two times (once per train-test pair). In addition, five randomly class-labeled datasets were generated from each trainset and used to train the BNC, which was then tested on the corresponding, non-randomly labeled, testset pair (i.e., held out GT group).

Within a general classification theory framework, classification performance evaluation of BNC’s was used as a basis for the evaluation of the feature subsets’ predictive usefulness and association to RR/SR response. Classification evaluation^[[4]](#footnote-4)^ of learned BNC’s was based on the classification accuracy (CA), precision, F measure and mean absolute error (MAE) metrics using the following formulas,

$$CA=\frac{TP+TN}{TP+TN+FP+FN}$$

$$Precision=\frac{TP}{TP+FP}$$

$$F measure=2\left( \frac{\left( \frac{TP}{TP+FP} \right)\left( \frac{TP}{TP+FN} \right)}{\left( \frac{TP}{TP+FP} \right)+\left( \frac{TP}{TP+FN} \right)} \right)$$

$$MAE=\frac{|p_{1}-a_{1}|+\cdots+|p_{n}-a_{n}|}{n}$$

where, $TP$ is the number (no.) of true positives; $TN$, the no. of true negatives; $FP$, the no. of false positives; and $FN$, the no. of false negatives; $p$ is the numerical value of the prediction for the *i*th test instance, and $a$ is the actual value of the *i*th test instance.

**2. Artificial neural network (ANN) classification method**: To further evaluate RR-/SR-specific association of a feature subset, we used an ANN technique based on the self-organizing tee algorithm (SOTA) ^12,13^. SOTA is a self-organizing/self-growing ANN for unsupervised clustering, which can generate a ‘distribution preserving’ binary tree that can adapt to the input data and reveal the natural structure relationships in data.

Briefly, HBV quasispecies sequences (N=954) were transformed into numerical vectors representing the physicochemical profiles of HBV sequences. Physicochemical profiles were based on physicochemical values ^14^ of nt’s. Numerical vectors were generated by concatenating physicochemical values of nt’s using the following formula,

$$V_{z}=k_{1}\left\{ X_{1},X_{2},X_{3},X_{4},X_{5} \right\}+\cdots+k_{16}\left\{ X_{1},X_{2},X_{3},X_{4},X_{5} \right\}$$

where, $V_{z}$ is the numerical vector representation of the physicochemical profile of an HBV sequence variant and $\{X_{1},X_{2},X_{3},X_{4},X_{5}\}$ numeric physicochemical values for the nt occupying a $k$ site. Physicochemical values: $X_{1}$ is phobicity; $X_{2}$, polarity; $X_{3}$, dipole moment; $X_{4}$, surface area and $X_{5}$, stacking area ^14^.

The SOTA ANN was constructed^[[5]](#footnote-5)^ in a series of cycles during which input physicochemical profiles was presented multiple times to neurons in the network. After each presentation, the neurons that are more closely associated to the profiles are updated to further closely match input profiles. At the end of each cycle, only the neurons with the highest resource^[[6]](#footnote-6)^ can generate daughter neurons, each adapted to the profiles representing the mean of the ancestor. Cycles continued until the level of variability in one, or more, neurons reached a predefined resource value (defined as the mean value of the distances between a neuron and the physicochemical profiles associated with it). Here, the SOTA ANN was implemented using the SOTA module available in KNIME software (v2.4) ^15^.

**SI Figures**

**Figure S1**


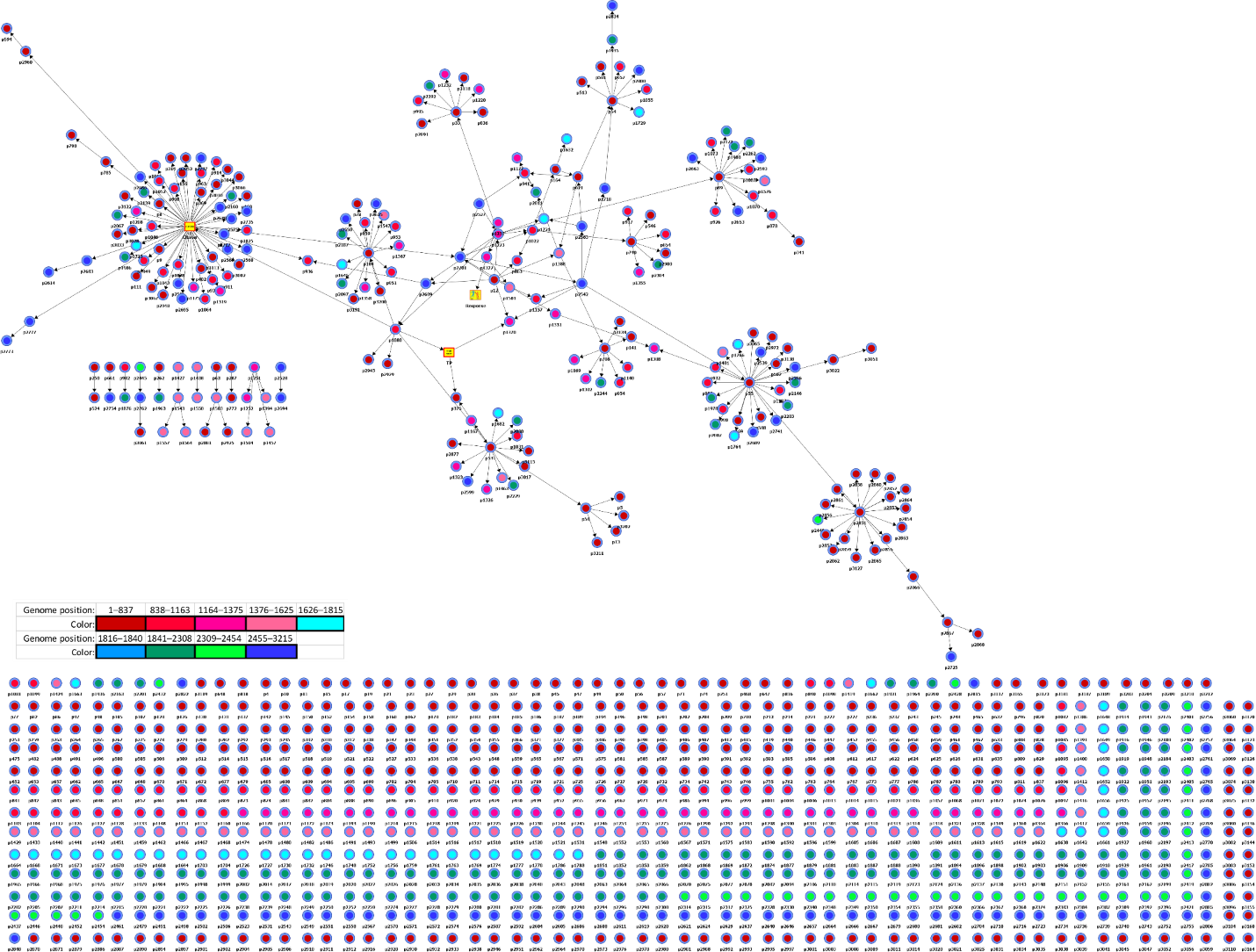


**Figure S1**. **Bayesian network (BN) of HBV GT C genomes**. The BN graph divides into a major 215-variable component comprised of 212 polymorphic nt sites (round nodes) and three “target” variables (response, time point (TP) and phylogenetic cluster (Cluster)– square nodes in yellow). Additionally, 30 nt sites divided into 11 minor components of up to 5 variables each. Arcs (N=251) in the graph represent strong relationships between variables. The remaining 778 polymorphic nt sites were not observed to have dependency relationships at the structural coefficient (SC) threshold ≥ 1 (unconnected nodes at bottom of the figure). BN was derived using HBV whole genome quasispecies (N=799) collected from TDF-treated immunocompetent patients (N=6) with chronic HBV/C infection. Node coloring based on representation of nine genomic regions (genome positions in parenthesis): overlapping S–P genes (1-837), RT domain (838-1163), RNAse H domain (1164-1375), overlapping RNAse H–X (1376-1625), X gene (1626-1815), overlapping X–C genes (1816-1840), C gene (1841-2308), overlapping C–P genes (2309-2454) and Terminal protein domain (2455-3215). Genome position numbering is based on GenBank reference sequence AY233278.

**Figure S2**


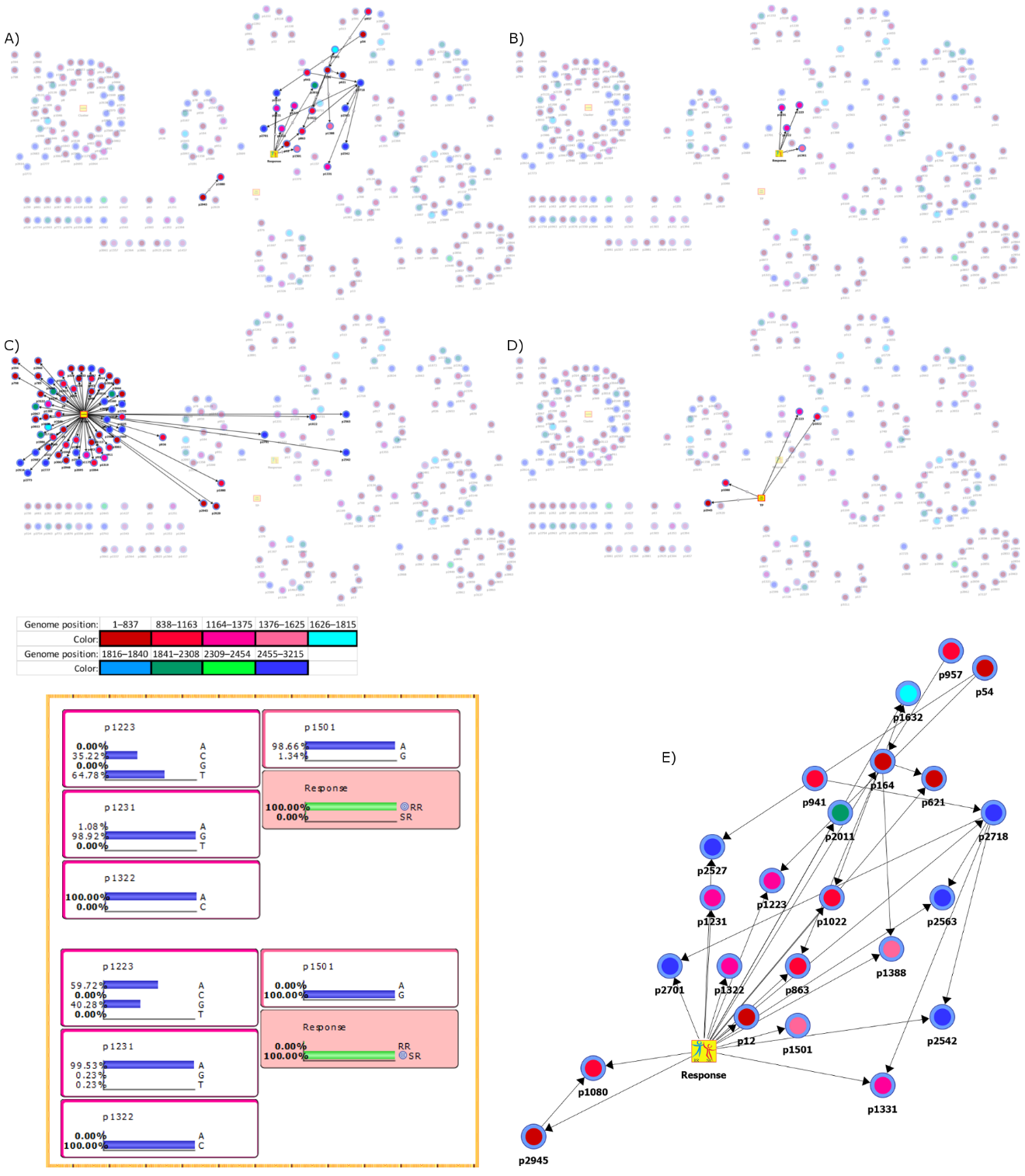


**Figure S2**. **Polymorphic Nt sites in HBV GT C genomes with strong target-specific association.** Shown are graphical representations of the target analysis done on the major 215-variable BN component (shown in Fig. 1 in manuscript). Round nodes in the graph represent nt sites and square nodes represent the “target” variables: response, phylogenetic clustering (Cluster) and time point (TP). Coloring of round nodes is based on genome positions (denoted in color legend). Genome position numbering is based on GenBank reference sequence AY233278. In graphs A thru C, visible nodes represent important polymorphic nt sites and visible arcs represent strong and important relationships (dependencies) as measured by Kullback–Leibler (KL) divergence ^8^ (details in SI Methods). Here, determination of the significance of strength of relationships was based on a KL-divergence threshold of ≥0.6837. A) 23 nt sites showed strong association to the response variable (KL values = 0.6837–0.9865). B) Among the 23 nt sites, four sites (genome positions 1223, 1231, 1322 and 1501) were members of the Markov blanket ^16^ and highly predictive of the RR/SR status. In position 1322, adenine was exclusively found in RR-associated variants, while cytosine was exclusive to SR-associated variants. Similarly, in position 1223, presence of cytosine/thymine and of adenine/guanosine was exclusive to RR- and SR-associated variants, respectively. Nt frequency distributions (denoted by percentages (%) in blue bars) at these four nt sites, specifically in relation to the target RR or SR status (green bars), are shown in the orange inbox. C) Among the 23 nt sites, six sites (genome positions 1022, 1080, 2542, 2563, 2701 and 2945) were also found strongly associated (KL values= 0.8205–0.9555) to the phylogenetic clustering variable (Fig. 1C in manuscript). D) None of the 23 nt sites were found to have important association to the time point variable. Visible nodes represent unimportant variables (nt genome positions: 1020, 1080, 1223 and 2945) and visible arcs represent weak (insignificant) relationships (KL ≤ 0.0893). E) Graph shows an enlarged depiction of the network comprising the response and the 23 nt site variables (graph A).

**Figure S3**


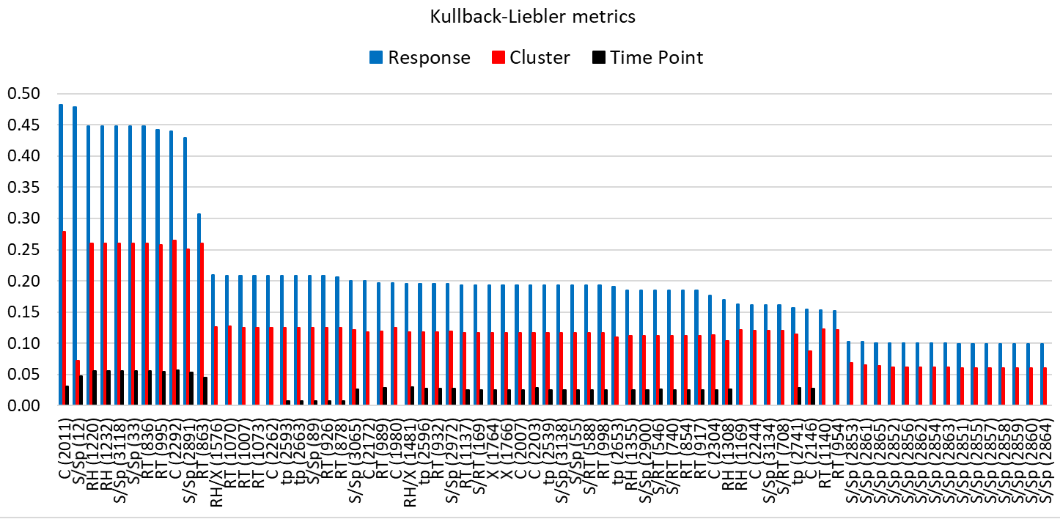


**Figure S3**. **Polymorphic** N**t sites in HBV/C genomes with significant association to TDF-associated responses**. Bar plot shows kullback-Liebler (KL) values (x-axis) of 72 polymorphic nt sites (y-axis) corresponding to the target variables response, phylogenetic cluster and time point (TP). Nt sites from the Sp (N=26), RT (N=5) and the RNAse H domains of the P gene, which overlapped with sites of the S gene (N=31) and the X gene (N=2), as well as, non-overlapping sites in the RT (N=16), RNAse H (N=5), terminal protein domains of the P gene (N=6), the C gene (N=10), and X gene (N=2) were found to have small but significant (p<0.001) association to the response variable. The largest continuous stretch of sites (N=15) associated to the response variable was contributed by genome positions 2851-2865 (corresponding to Sp domain, overlapping P and S genes). Target analysis was conducted on the major BN 215-variable component in the same manner as for data shown in Fig. S2. KL values was obtained from BN target analysis (Fig. S2). BN nodes whose KL values with respect to the response variable are significantly (p<0.001) stronger to the other two target variables are shown. Members of the RR/SR Markov blanket (nodes representing genome positions 1223, 1231, 1322 and 1501 in Fig. S2B) were removed from BN prior to analysis. Nt sites (y-axis) are annotated by genomic region and by genomic position (in parenthesis). Overlapping regions indicated by “/” symbol. Genome position numbering is based on GenBank reference sequence AY233278.

**Figure S4**


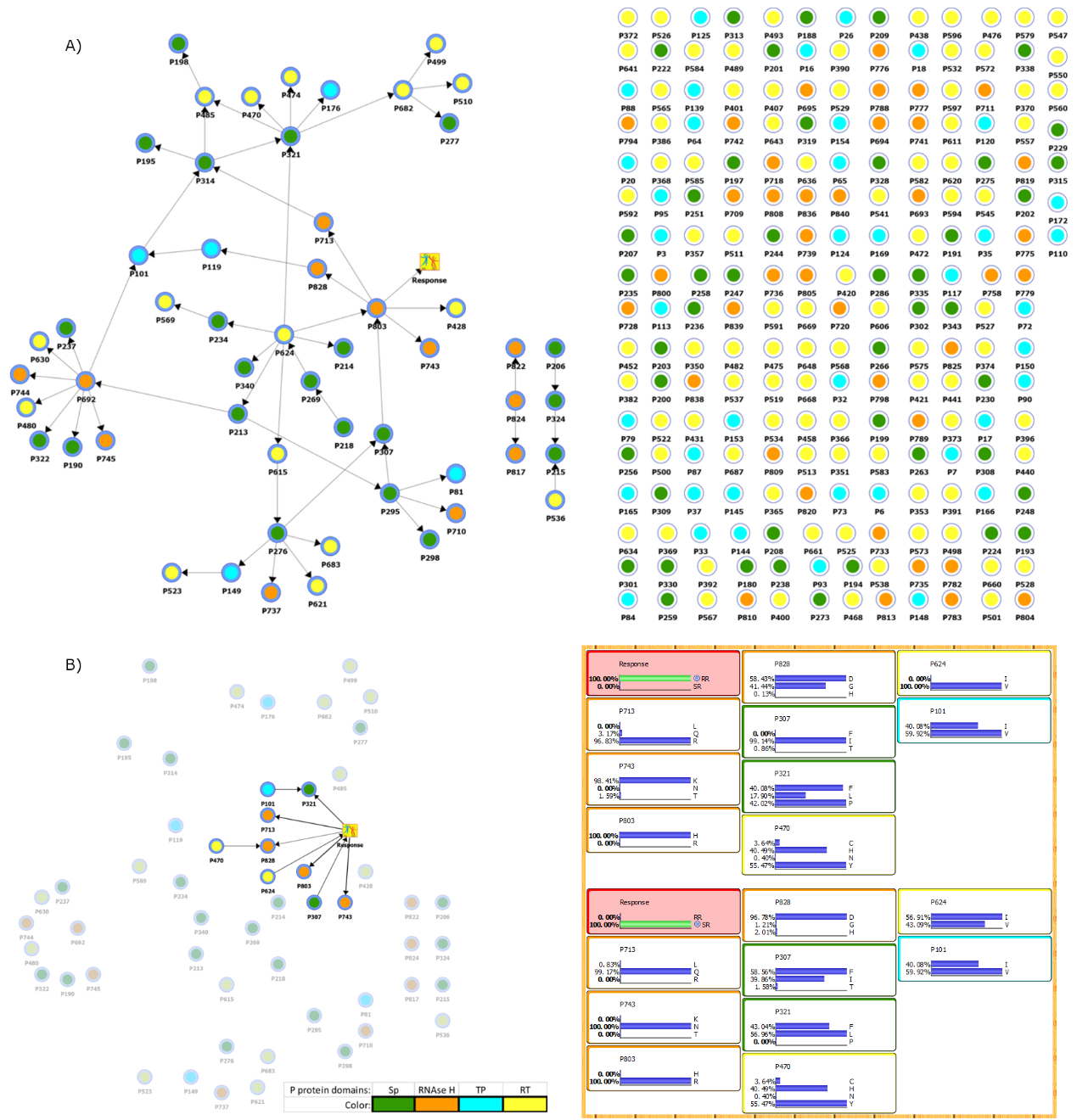


**Figure S4. BN of the HBV GT C polymerase protein.** The BN model was constructed using unique 843-long amino-acid (aa) quasispecies sequences of the polymerase protein (N=247) collected from patients six patients (P1-P6) prior to start-of-treatment (at baseline). A) The BN graph divides into a major 48-varaible component and into two minor 3- and 4-variable components. Polymorphic aa sites (N=211) not observed forming dependency relationships at the threshold of significance (SC≥0.95 and p<0.001) are shown as unconnected nodes at right of the figure. Nodes representing aa sites are colored according to the four polymerase domains (denoted in color bar). Spacer (Sp), reverse transcriptase (RT), ribonuclease H (RNase H) and the terminal protein (tp) domains were observed contributing to the major 48-varable component with 21, 16, 12 and 5 aa sites, respectively. B) Among the set of polymorphic aa sites comprising the major 48-variable network, 7 sites (Sp sites 307 and 321; RT site 624, and RNAse H sites 713, 743, 803 and 828) – shown as visible nodes in graph– were identified through target analysis as members of the Markov blanket and strongly (KL≥0.824) associated to RR/SR states encoded in the response variable (square node). Aa frequency distributions (blue bars) at these 7 sites respective to the RR and SR states (green bars) are shown in the inbox. Polymerase position numbering is based on GenBank reference sequence AF458665.1.

**Figure S5**


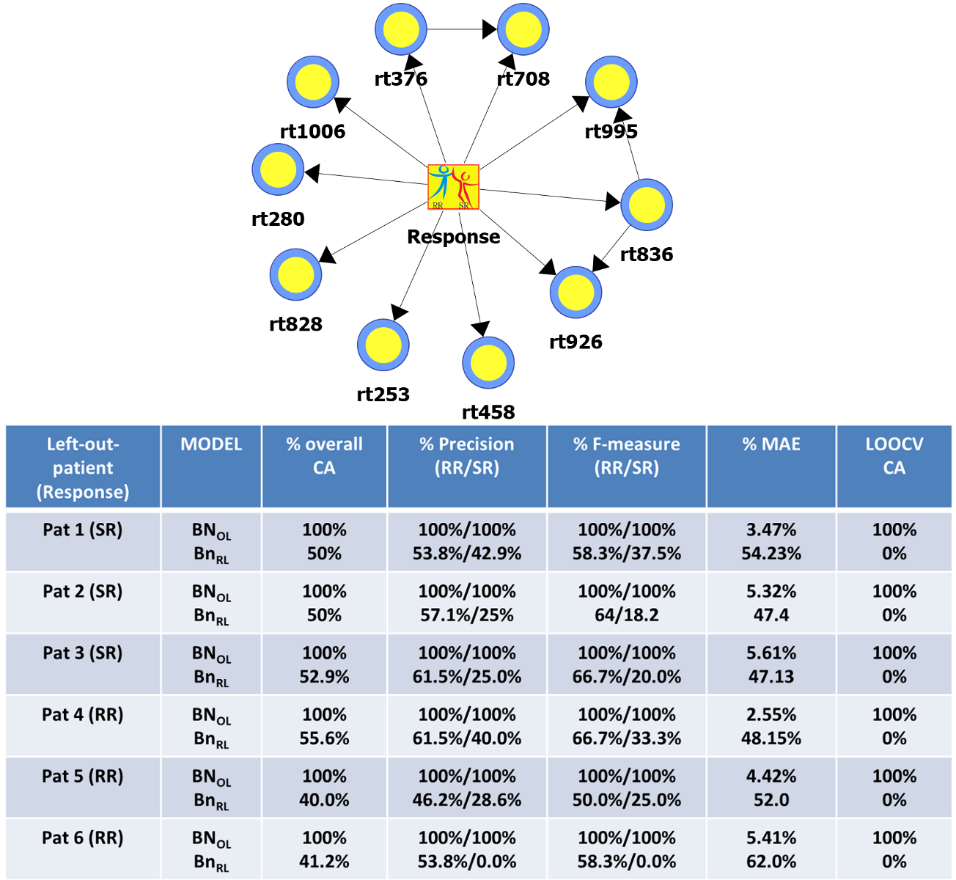


**Figure S5.** **RR/SR association with nt substitutions in RT HBV/C genomes.** Shown is a graphical representation of a Bayesian network classifier (BNC) and its classification performance for association of RT HBV/C variants to RR/SR states. Quasispecies sequences (N=247) of the RT domain of polymerase (genome positions 132-1166) from six HBV/C infected patients (P1-P6) were sampled prior to start-of-treatment (at baseline). The CFS feature selection algorithm ^17^ was applied to the RT sequence dataset to derive the best minimum subset of features (or attributes), which was found to be comprised of 10 nt sites (Merit=0.527). The 10 nt sites were then used to construct the BNC (SC≥0.70) to establish RR/SR and inter-site dependencies –details in SI Methods. Node/site numbering is based on GenBank sequence AF458665.1. The table below the graph shows classification performance of the BNC in leave-one-out cross-validation (LOOCV) repeated 10 times. Briefly, BNC were trained on HBV/C RT variants from five patients by 10-fold CV. The trained BNC model was then evaluated on the RT variants of the left-out patient. Experiments were performed using two BNC models: one trained on a dataset with the correct observed RR/SR labeling (BN_OL_ model) and the other trained on a randomly RR/SR labeled dataset (BN_RL_ model). The percentage (%) values for RR/SR classification accuracy (CA), precision, F-measure and mean absolute error (MAE) of the respective BNC model represent overall values of ten LOOCV repeats –details in SI Methods.

**Figure S6**


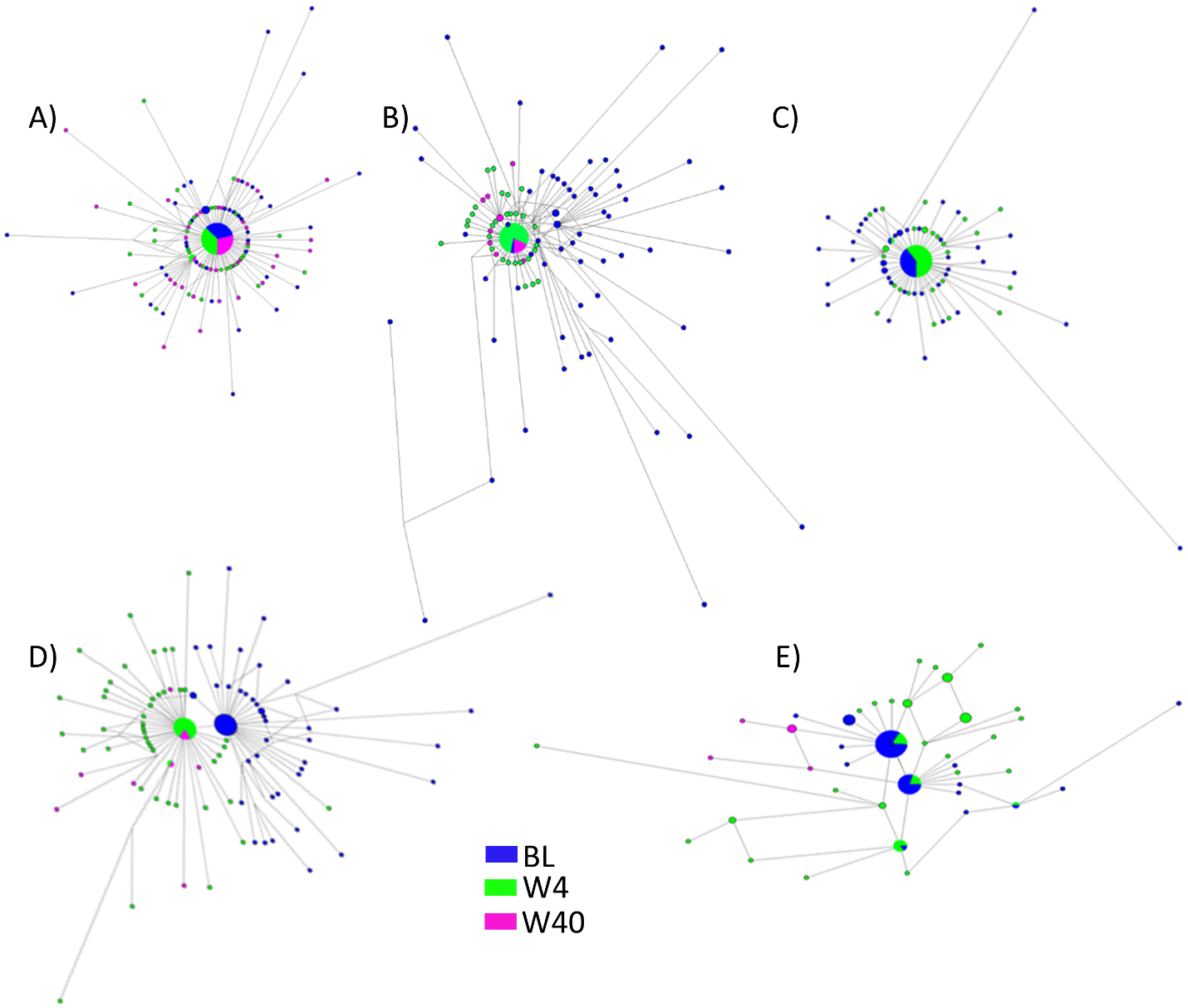


**Figure S6. RT strains selected for protein tertiary structure modeling.** Shown are the median joining networks (MJN) of HBV/C and HBV/B full-genome quasispecies. HBV/C strains from: A) SR patient P1, B) SR patient P2 and C) RR patient P5. HBV/B strains from: D) SR patient P8 and E) RR patient P9. RT sequences of the major HBV strain (large center node in graph), were selected to construct the 3-D structures of HBV RT protein variants. P8 presented with two major strains, which, however, shared identical RT protein sequences. Nodes in the MJN graph represent full-genome HBV variants and node size represent variant frequency. Node colors represent the three time points of sampling: baseline (BL) – prior to start-of-treatment – and week (W) 4 and 40 during TDF treatment (denoted in color legend). MJNs were generated using the Network software v4.112 ^18^.

Figure S7


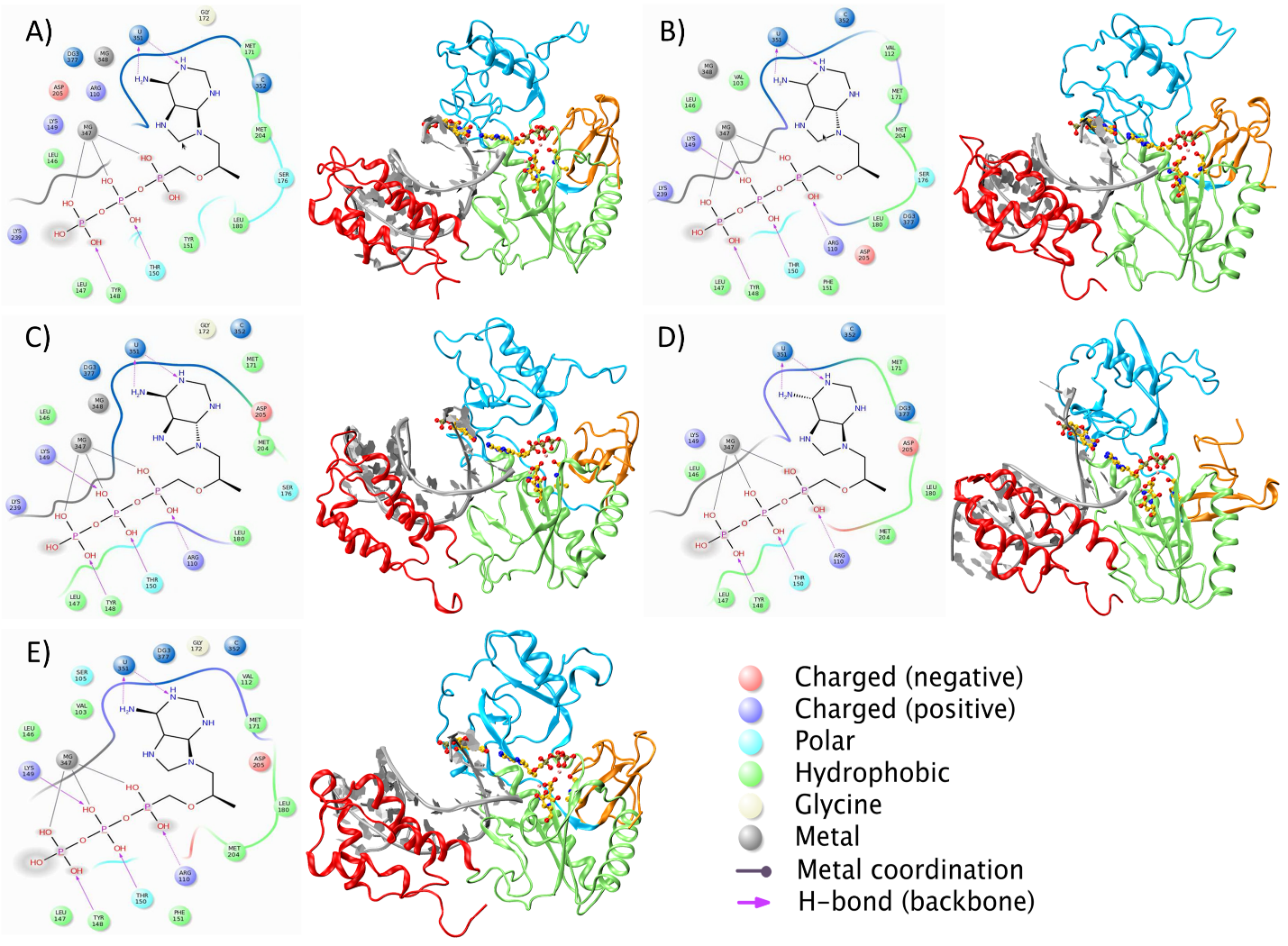


**Figure S7.** **TFV-DP and HBV RT protein-ligand interaction**. Shown are schematic 2-dimensional (2D) ligand interaction diagrams (on the left) and corresponding predicted 3D structures of protein-ligand complexes (on the right). Ligand interaction diagrams of Tenofovir Diphosphate (TFV-DP) in complex with five distinct HBV RT proteins with the native hybrid DNA-RNA substrate in place. A) RR P9 (HBV/B), B) RR P5 (HBV/C), C) SR P1 (HBV/C), D) SR P8 (HBV/B) and E) SR P2 (HBV/C). Nodes in diagrams represent residue sites in RT or nt sites (blue nodes) in DNA/RNA template strand or metals (grey nodes). Chemical structures in diagrams represent the TFV-DP substrate. Contour lines represent the protein interface, and H-bonds and metal interactions are respectively represented as directed or undirected vertices. Coloring of nodes representing aa sites and contour lines is based on physico-chemistry (shown in legend). Grey shading around –OH groups denotes solvent accessibility/exposure. 3D models: the RT protein structure is colored by domains: fingers, in cyan and gold; palm, in green, and thumb, in red. TFV-DP ligand is depicted as ball-and-stick (cpk color mode) and DNA/RNA ligand as cartoon (grey color). Visual rendering of 3D protein-ligand complexes was done using the VMD software ^19^. Full description of the protein-ligand modeling approach used is found in our previous study ^20^. Summary of ligand interaction findings is shown in Table S4.

**Table S1**. **Overall classification performance of 16nt polymorphic sites in LOOCV-GT**

| **GT–BN model** | **% Overall CA on training set** | **% Overall CA on test set** |
| --- | --- | --- |
| C–BNC_OL_ | 100%^‡^ | 99.4% |
| C–BNC_RL_ | 49.3%^§^ | 43.8% |
|  |  |  |
| B&E–BNC_OL_ | 100%^‡^ | 99.8% |
| B&E–BNC_RL_ | 52.4%^§^ | 49.7% |
|  |  |  |
| Shown are overall classification accuracy (CA) (95% CI 99.4%-100%) of Bayesian network classifier (BNC) models on leave-one-genotype-out (LOOCV-GT) using nt information from 16 polymorphic sites spanning different regions of the genome (see Table 1 in manuscript).  C–BNC_OL_: BNC model trained with dataset of HBV/C quasispecies (N=611) sampled from three SR patients (P1–P3) and three RR patients (P4–P6) and tested on dataset of HBV quasipecies (N=343) collected from RR patients (P7 & P9) and an SR patient (P10).  B&E–BNC_OL_: model trained with dataset of HBV/B and HBV/E quasispecies (N=343) from SR patients (P8 & P10) and RR patients (P7 & P9) and tested on the dataset of HBV/C quasispecies (N=611) from RR and SR patients (P1–P6).  C–BNC_RL_ and B&E–BNC_RL_ models were trained on randomly RR/SR labeled datasets and then tested on the correctly observed RR/SR labeled left-out samples (details in SI Methods).  ^‡^ Represents an averaged (Avg.) CA value over 10-fold CV tests.  ^§^ Represents an Avg. CA value over 5 repeated 10-fold CV tests. | | |

**Table S2**. **Overall classification performance of 16nt polymorphic sites in LOOCV-P**.

| Patient (no. of variants sampled) | BN model | % overall CA^‡^ (no. of variants in training set) | % CA on testset |
| --- | --- | --- | --- |
| Patient 1 (109) | BN_OL_ | 100 (845) | 100 |
|  | BN_RL_ | 50.3^§^ | 59.8 |
| Patient 2 (98) | BN_OL_ | 100 (856) | 100 |
|  | BN_RL_ | 49.4^§^ | 39.0 |
| Patient 3 (103) | BN_OL_ | 99.8 (851) | 100 |
|  | BN_RL_ | 52.1^§^ | 21.9 |
| Patient 4 (112) | BN_OL_ | 100 (842) | 100 |
|  | BN_RL_ | 58.3^§^ | 20.9 |
| Patient 5 (67) | BN_OL_ | 100 (887) | 100 |
|  | BN_RL_ | 53.4^§^ | 57.3 |
| Patient 6 (122) | BN_OL_ | 100 (832) | 100 |
|  | BN_RL_ | 57.0^§^ | 2.1 |
| Patient 7 (110) | BN_OL_ | 99.8 (844) | 100 |
|  | BN_RL_ | 56.3^§^ | 2.5 |
| Patient 8 (94) | BN_OL_ | 100 (860) | 97.9 |
|  | BN_RL_ | 50.5^§^ | 95.3 |
| Patient 9 (47) | BN_OL_ | 100 (907) | 100 |
|  | BN_RL_ | 51.6^§^ | 78.7 |
| Patient 10 (92) | BN_OL_ | 99.8 (862) | 100 |
|  | BN_RL_ | 50.5^§^ | 0.0 |
|  |  |  |  |
| Shown are overall classification accuracy (CA) (95% CI 99.4%-100%) of BNCs using nt information from 16 polymorphic sites (see Table 1 in manuscript). In the leave-one-patient-out (LOOCV-P) scheme, BNC models (BNC_OL_) were trained on HBV quasispecies sequence data from 9 patients and then tested on dataset of HBV quasispecies sampled from the left-out patient (details in SI Methods).  BNC models (BNC_RL_) were trained on randomly RR/SR labeled datasets and tested on the correctly labeled dataset from the left-out patient (repeated 5 times per patient).  ^‡^ Represents an averaged (Avg.) value over 10-fold CV tests.  ^§^ Represents an Avg. value over 5 repeated 10-fold CV tests. | | | |

**Table S3. RR/SR association with aa substitutions in RT HBV/C proteins**.

| **Protein position^‡^** | |
| --- | --- |
| **Polymerase protein** | **RT domain** |
| 350, 357, 369, 392, 428, 475, 480, 485, 493, 499, 537, 560, 569, 579, 585, 592, 621, 624, 634, 669, 682 and 683 | 4**^c^**, 11, 23, 46, 82**^a, c^**, 129, 134**^c^**, 139**^a, c^**, 147**^b^**, 153**^a, c^**, 191**^a,b^**, 214, 223**^a, c^**, 233**^a,c^**, 239**^b^**, 246, 275**^c^**, 278**^c^**, 288**^b^**, 323, 336**^c^** and 337**^c^** |
|  |  |
| Shown are 22 aa polymorphic sites comprising the best subset features found with strong association (Merit=0.511) to the RR/SR response, which was obtained by applying the CFS feature selection algorithm ^17^ to a dataset comprised of the 345-long aa RT protein quasispecies (N=247) collected from 6 patients prior to start-of-treatment (at baseline) – details in SI Methods.  These 22 aa sites were found to be useful for RR/SR association of HBV RT protein variants, as determined by BNC models (details in SI Methods). The observed overall performance achieved by a 22 aa-based BNC was: classification accuracy (CA)=98.8%, Precision=99.0%, F-measure=99.0% and mean absolute error (MAE) =1.0%. Meanwhile, the overall classification performance of a BNC model trained on randomly RR/SR labeled datasets was observed to deteriorate close to expected values (CA=53.4%, Precision=54.0%, F-measure=53.0% and MAE=49.0%).  **^‡^** Position numbering in polymerase and corresponding RT positions based on reference sequence: GenBank accession number AF458665.1.  **^a^** Sites with reported association to HBV drug resistance (discussed in Results Section of manuscript).  **^b^** Sites identified here as potential effector sites associated with the TVF-DP ligand interaction with the RT receptor (Table S4).  **^c^** Variables in BN observed to establish significant (KL≥0.1≤0.4; p≤0.001) relationships with the response variable (Fig. S4). | |

**Table S4. Summary of TFV-ligand interactions (shown in Fig.S7)**

|  |  | **TFV-DP ligand interactions ^b^** | | | | |
| --- | --- | --- | --- | --- | --- | --- |
| **Associated Response** | **HBV RT^a‡^**  **[HBV GT]** | **Residue H-bonds^§^** | **Metal** | **RNA H-bonds** | **Contacts with YMDD sites^§^** | **Contacts with sites in RT-BNC ^c§^** |
| SR | RT1 [C] | Y148 (1), T150 (1), R110 (1) and K149 (1) | Mg^+2^ (3) | U (2) | M204, D205 | L147, K239 |
| SR | RT2 [C] | Y148 (1), T150 (1), R110 (1) and K149 (1) | Mg^+2^ (3) | U (2) | M204, D205 | L147, K239 |
| RR | RT5 [C] | Y148 (1), T150 (1), R110 (1) and K149 (1) | Mg^+2^ (3) | U (2) | M204, D205 | L147, K239 |
|  |  |  |  |  |  |  |
| RR | RT9 [B] | Y148 (1), T150 (1), R110 (1) and K149 (1) | Mg^+2^ (3) | U (2) | M204, D205 | L147, K239 |
| SR | RT8 [B] | Y148 (1), T150 (1), R110 (1) and K149 (1) | Mg^+2^ (3) | U (2) | M204, D205 | L147, K239 |
|  |  |  |  |  |  |  |
| List of residue/RNA hydrogen bonds (H-bonds), metal coordination coupling and residue contacts that persist throughout the unconstrained MD (120 ns) simulation – details in ^20^. Values in parenthesis represent number of H-bonds or number of metal couplings.  ^a^ HBV RT variants enumerated based on patient ID numbering used here.  ^b^ Capitalized letters denote: tyrosine (Y), threonine (T), arginine (R), lysine (K), asparagine (D) and methionine (M) residues, RNA nucleic acid uracil (U) and divalent magnesium cation (Mg^2+^).  ^c^ Residue sites comprising the RT 22 aa-based BNC (RT-BNC) (Table S3) that were mapped in the vicinity of the binding pocket (Fig. 5 in Manuscript).  ^§^ RT residue numbering based on reference sequence: GenBank accession number AF458665.1.  **^‡^** RT protein variants RT1, RT2, RT5, RT9 and RT8 were respectively denoted as RT1, RT2, RT3, RT5 and RT4 in our previous study ^20^. | | | | | | |

**Table S5. Baseline demographics of ten immune tolerant patients**

| **Patient ID** | **HBV**  **Genotype** | **HBeAg Status** | **Response Status^a^** | **HBV DNA**  **(log10 IU/mL)** | **ALT**  **(U/L)** | **Gender^b^** | **Age** | **Race^c^** | **Treatment** |
| --- | --- | --- | --- | --- | --- | --- | --- | --- | --- |
| P1 | C | Positive | SR | 9.73 | 19 | M | 29 | AS | TDF |
| P2 | C | Positive | SR | 9.67 | 5 | M | 20 | AS | TDF |
| P3 | C | Positive | SR | 9.63 | 21 | M | 38 | AS | TDF |
| P4 | C | Positive | RR | 9.57 | 32 | M | 35 | AS | TDF |
| P5 | C | Positive | RR | 9.69 | 17 | M | 28 | AS | TDF |
| P6 | C | Positive | RR | 9.46 | 26 | F | 55 | AS | TDF |
| P7 | B | Positive | RR | 9.41 | 17 | F | 30 | AS | TDF |
| P8 | B | Positive | SR | 9.65 | 41 | M | 46 | AS | TDF |
| P9 | B | Positive | RR | 9.41 | 35 | M | 39 | AS | TDF |
| P10 | E | Positive | SR | 9.43 | 20 | F | 34 | Bl | TDF |
| Shown are demographics of the 10-patient cohort prior to start of TDF monotherapy, including the hepatitis B e-antigen (HBeAg) and HBV DNA level tests, and the Alanine Aminotransferase (ALT) levels.  **^a^** Abbreviations: Slow Response, SR; Rapid Response, RR.  **^b^** Abbreviations: Male, M; Female, F.  **^c^** Abbreviations: Asian, AS; Black, Bl. | | | | | | | | | |

References:

1 Jensen, F. *Bayesian Networks and Decision Graphs*. 3-30 (Springer, 2001).

2 Neapolitan, R. E. *Learning Bayesian Networks*. Vol. 1st 29-47 (Pearson/Prentice Hall, 2004).

3 Chickering, D. M., Heckerman, D. & Meek, C. Large-sample learning of Bayesian networks is NP-hard. *J Mach Learn Res* **5**, 1287-1330 (2004).

4 Bouckaert, R. in *Symbolic and Quantitative Approaches to Reasoning and Uncertainty* Vol. 747 *Lecture Notes in Computer Science* (eds Michael Clarke, Rudolf Kruse, & Serafín Moral) 41-48 (Springer Berlin / Heidelberg, 1993).

5 Rissanen, J. Stochastic Complexity and Modeling. *Ann Stat* **14**, 1080-1100 (1986).

6 Munteanu, P. & Bendou, M. in *2001 Ieee International Conference on Data Mining, Proceedings.* (eds N. Cercone, T. Y. Lin, & X. Wu) 417-424 (IEEE).

7 Jouffe, L. & Munteanu, P. in *Proceedings of the 10th International Symposium on Applied Stochastic Models and Data Analysis, Compiègne, France.* 591-596.

8 Kullback, S. & Leibler, R. A. On information and sufficiency. *Ann Math Stat* **22**, 79-86 (1951).

9 Korb, K. & Nicholson, A. *Bayesian Artificial Intelligence*. (Chapman & Hall/CRC Press, 2004).

10 Peng, C., Xiao, S., Nie, Z., Wang, Z. & Wang, F. Applying Bayes' theorem in medical expert systems. *IEEE ENG MED BIOL* **15**, 76-79 (1996).

11 Cooper, G. F. & Herskovits, E. J. A Bayesian method for the induction of probabilistic networks from data. *Machine Learning* **9**, 309-347, doi:10.1007/bf00994110 (1992).

12 Dopazo, J. & Carazo, J. M. Phylogenetic reconstruction using an unsupervised growing neural network that adopts the topology of a phylogenetic tree. *J Mol Evol* **44**, 226-233 (1997).

13 Herrero, J., Valencia, A. & Dopazo, J. A hierarchical unsupervised growing neural network for clustering gene expression patterns. *Bioinformatics* **17**, 126-136 (2001).

14 Guckian, K. M. *et al.* Factors Contributing to Aromatic Stacking in Water: Evaluation in the Context of DNA. *J Am Chem Soc* **122**, 2213-2222, doi:10.1021/ja9934854 (2000).

15 Berthold M.R. et al. KNIME: The Konstanz Information Miner. In: Preisach C., Burkhardt H., Schmidt-Thieme L., Decker R. (eds) Data Analysis, Machine Learning and Applications. Studies in Classification, Data Analysis, and Knowledge Organization. (Springer, Berlin, Heidelberg, 2008) https://doi.org/10.1007/978-3-540-78246-9_38

16 Pearl, J. *Probabilistic reasoning in intelligent systems: networks of plausible inference*. (Morgan Kaufmann Publishers Inc., 1988).

17 Hall, M. A. *Correlation-based Feature Selection for Machine Learning* Doctor of Philosophy thesis, The University of Waikato, New Zeland, (1999).

18 Bandelt, H. J., Forster, P. & Rohl, A. Median-joining networks for inferring intraspecific phylogenies. *Mol Biol Evol* **16**, 37-48, doi:10.1093/oxfordjournals.molbev.a026036 (1999).

19 Humphrey, W., Dalke, A. & Schulten, K. VMD: visual molecular dynamics. *J Mol Graph* **14**, 33-38, 27-38 (1996).

20 Xu, X. *et al.* Modeling the functional state of the reverse transcriptase of hepatitis B virus and its application to probing drug-protein interaction. *BMC Bioinform* **17 Suppl 8**, 280, doi:10.1186/s12859-016-1116-4 (2016).

1. The cycle of exploration in BN space continued until no further improvement in the MDL score was observed. [↑](#footnote-ref-1)
2. Manifestations refer to the treatment responses RR or SR observed in 10 HBV-infected patients. [↑](#footnote-ref-2)
3. Two train/test pairs: GT-C/GT-B&E and GT-B&E/GT-C. [↑](#footnote-ref-3)
4. Implemented in WEKA software (v3.17) (https://www.cs.waikato.ac.nz/ml/weka/). [↑](#footnote-ref-4)
5. This process did not require or took into account the RR/SR class labels associated with the input profiles. [↑](#footnote-ref-5)
6. ‘Resource’ is a measure of heterogeneity (herein, based on the cosine distance metric). A variability threshold=0.01 was used to restrict the growth of the SOTA ANN. [↑](#footnote-ref-6)
